# Supplementary material for: Myosin II Filament Dynamics in Actin Networks Revealed with Interferometric Scattering Microscopy
Source: Biophys J. 2020 Mar 4;118(8):1946–57. doi: 10.1016/j.bpj.2020.02.025 (PMC7175421; doi:10.1016/j.bpj.2020.02.025)
Supplement: Document S1. Supporting Materials and Methods, Figs. S1–S3, and Table S1 [file mmc1.pdf]

Biophysical Journal, Volume *118*

Supplemental Information

Myosin II Filament Dynamics in Actin Networks Revealed with Interferometric Scattering Microscopy

Lewis S. Mosby, Nikolas Hundt, Gavin Young, Adam Fineberg, Marco Polin, Satyajit Mayor, Philipp Kukura, and Darius V. Köster

Myosin II filament dynamics in actin networks revealed with interferometric scattering microscopy

Lewis S. Mosby[1,2]*, Nikolas Hundt[3,Ɨ,]*, Gavin Young[3], Adam Fineberg[3], Marco Polin[1,2], Satyajit Mayor[4,5,#], Philipp Kukura[3#], Darius V. Köster[1,6#]

1 Centre for Mechanochemical Cell Biology, University of Warwick, Coventry CV4 7AL, UK

2 Physics Department, University of Warwick, Coventry CV4 7AL, UK

3 Physical and Theoretical Chemistry Laboratory, Department of Chemistry, University of Oxford, South Parks Road, Oxford OX1 3QZ, UK

4 National Centre for Biological Sciences, Tata Institute for Fundamental Research, GKVK, Bellary Road, Bangalore 560065, India

5 Institute for Stem Cell Biology and Regenerative Medicine, Bangalore 560065, India

6 Division of Biomedical Sciences, Warwick Medical School, University of Warwick, Coventry CV4 7AL, UK

* equal contribution

#corresponding authors: mayor@ncbs.res.in, philipp.kukura@chem.ox.ac.uk, d.koester@warwick.ac.uk

Ɨ current address: Department of Cellular Physiology, Biomedical Center, Ludwig-Maximilians-Universität München, Großhaderner Str. 9, 82152 Planegg/Martinsried, Germany

Supplementary material

Detection of myosin II filaments

We use two criteria for the detection of a bound myosin filament: particles have to be above an intensity threshold, and they have to display a minimal area characteristic for a single myosin filament oriented along the focal plane. This ensures that the probability to detect a myosin filament that is diffusing freely and not binding to F-actin is very low.

Considering an unbound myosin filament, it would move out of radius *r* = 200nm sphere within <10 ms, which would not generate enough signal to be considered by our tracking algorithm.

This estimate is based on taking the average time for a diffusing particle to travel from the center to a point on a sphere with radius *r*: $\bar{t} = \frac{r^2}{6D}$

And with $D = \frac{\mathrm{k_B}T}{6\,\pi\gamma R}$, where $\gamma = 0.89\ 10^{-3}\ \mathrm{kg\ s^{-1}\ m^{-1}}$ is the viscosity of water at 23 °C and $R\ =\ 250\ \mathrm{nm}$ is the radius of a sphere to represent a myosin filament, we get

$$\bar{t} = \frac{(0.2\ \mu\mathrm{m})^2}{6\ \times\ 1.48\ \mu\mathrm{m}^2\mathrm{s}^{-1}} = 6.7\ \mathrm{ms}$$

And if we take into account the elongated shape of myosin, with the long semi-axis $b\ =\ 300\ \mathrm{nm}$, a short semi-axis $a\ =\ 50\ \mathrm{nm}$ and $D = \frac{\mathrm{k_B}T\ \ln(2b/a)}{4\,\pi\gamma\ b}$, we get

$$\bar{t} = \frac{(0.2\ \mu\text{m})^2}{6\ \times 3.31\ \mu\text{m}^2\text{s}^{-1}} = 3\ \text{ms}$$

Mobility of actin filaments and slippage of myosin II filaments on supported lipid bilayers

Here, we are considering the possibility that the motor action of a myosin II filament would lead to a slippage of the actin filament resulting in a reduced net velocity of the myosin filament. For this, we compare the friction of the actin filament created by the actin-membrane linker and compare it to the friction that myosin II filaments experience in aqueous solutions.

Similar to considerations described in (30), we can estimate the frictional force on the actin-membrane linker by using the Einstein-Smoluchowski relation, where the drag coefficient is related to its diffusivity D_{HKE} (= 1 $\mu m^2 s^{-1}$) by:

$$\gamma_{HKE} = \frac{\mathrm{k_B}T}{D_{HKE}}$$

The drag force on a single actin filament could then be estimated as:

$$F_{actin} = \ L_{actin}\, \rho_{HKE}\gamma_{HKE}\ v_{actin} = L_{actin}\, \rho_{HKE}\frac{\mathrm{k_B}T}{D_{HKE}}\ v_{actin}$$

If we consider an actin filament of L_{actin} = 5 µm length, a linear membrane-actin linker density of ρ_{HKE} = 30 μm^{-1} and a maximum velocity of v_{actin} = 400 nm s^{-1} (if the myosin would stand still and move actin at maximum speed) the drag force would be

$$F_{actin}^{max} = 0.25\ \text{pN}$$

with k_B = 1.38 10^{-23} JK^{-1} and T = 295K.

In contrast, the drag force that acts on a myosin filament when it moves through an aqueous solution, can be estimated using the drag coefficient of a cylindrical object of length L and radius r moving parallel to the surface in a distance h:

$$\gamma_{\text{myo}} = \frac{2\pi\eta \text{L}_{\text{myo}}}{\ln(^{2\text{h}}/_{\text{r}_{\text{myo}}})}$$

And the drag force for the myosin filament would be

$$\text{F}_{\text{myo}} = \ \gamma_{\text{myo}}\text{v}_{\text{myo}} = \frac{2\pi\eta \text{L}_{\text{myo}}}{\ln(^{2\text{h}}/_{\text{r}_{\text{myo}}})}\ \text{v}_{\text{myo}}$$

With η = 10^{-3} Pa s (water), L_{myo} = 550 nm, h = 60 nm, r_{myo} = 30 nm, and v_{myo} = 400 nm s^{-1} the drag force is

$$\text{F}_{\text{myo}} = 1\ \text{fN}$$

That means that the drag on the myosin filament is 250 times smaller than the drag on the actin filament. This explains why myosin filaments move along single actin filaments without causing any significant displacement of actin filaments and why changes in actin filament organization only occur when myofilaments bind to multiple actin filaments, resulting to higher friction on myosin II filament motion and force transduction to the actin network.

Video legends, supplementary figures and tables

Video 1 Movie recorded with a 445 nm laser iSCAT system showing the formation of an SLB by landing and fusion of single uni-lamellar vesicles on a glass slide. The reduced contrast at the end of the video indicates the presence of the SLB as it smoothens the glass roughness.

Video 2 Movie showing the increase in interferometric scattering when an actin filament lands on top of another (Fig. S1E, F)

Video 3 Movie showing diffusion of a 1 µm long actin filament within a membrane-bound actin network (removed by median filtering) (Fig. 1E, F and Fig. S1H)

Video 4 Movie showing diffusion of a 4 µm long actin filament within a membrane-bound actin network (removed by median filtering) (Fig. 1G and Fig. S1I)

Video 5 Movie showing the transition of the acto-myosin network from a remodeling to a contractile state upon depletion of ATP over time (Fig. S2A, B).

Video 6 Movie showing the release of myosin II filaments from actin after addition of fresh ATP (100 µM) indicating that the contractile state of myosin II filaments in this set up is reversible.

Video 7 Movie showing myosin II filament motion along actin filaments (Fig. S2C, D). Scale bar 1 µm.

Video 8 Movie showing tracking of a single myosin filament (Fig. 3A, B; S3 A-C). Scale bar 1 µm.

Video 9 Movie showing the flow of myosin II filaments into and within an acto-myosin cluster after subtraction of the time median (Fig. 4 A, B). Scale bar 2 µm.

Figure S1

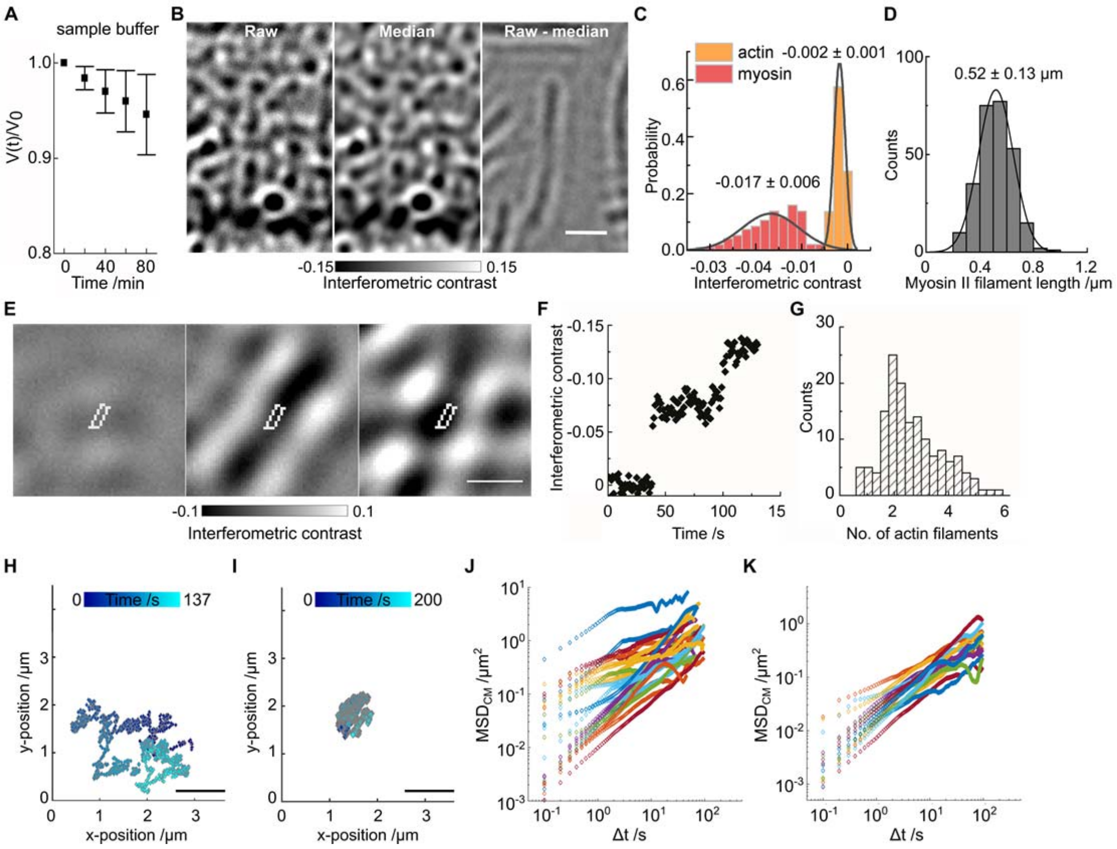

Figure S1, Detection and identification of actin and myosin II filaments: A) Graph illustrating the typical loss of buffer due to evaporation from the experimental chambers at room temperature; data points represent average ± standard deviation of three independent experiments. **B)** Example of median filter to visualize individual actin filaments landing afresh or moving within a static actin network; the median of the image sequence is subtracted from the raw images; 445 nm laser set-up, scale bar: 1 µm. **C)** Histogram of actin filament and myosin II filament contrast measurements with the 635 nm laser iSCAT set-up; actin filaments: N = 352 measurements along N_{fil} = 15 filaments and N_{myoII} = 94; black lines depict fit of normal distribution. **D)** Histogram of myosin II filament length measurements with the 635 nm laser iSCAT set-up; black line depicts fit of normal distribution; N = 269. **E)** Image sequence of an actin filament landing on top of another imaged with the 445 nm iSCAT setup. **F)** Corresponding measurement of the interferometric contrast of the region depicted by the white box in (E) showing a stepwise increase of the signal corresponding to one actin filament landing on top of another. **G)** Histogram of the actin layer thickness in a typical experiment based on background-subtracted, local interferometric contrast measurements divided by the average contrast value of an individual actin filament (C) (N=149). **H)** Center of mass trajectory of a 1 µm long actin filament (Fig. 1E, F; Video 3) over time (from dark blue to cyan: 0- 137s), scale bar: 1 µm. **I)** Center of mass trajectory of a 4 µm long actin filament (Fig 1G; Video 4) confined by the surrounding actin network (from dark blue to cyan: 0-200 s), scale bar: 1 µm. **J, K)** Combined plot of MSDs of actin filaments shorter than 1.5 µm (**J**) or longer (**K**).

Figure S2

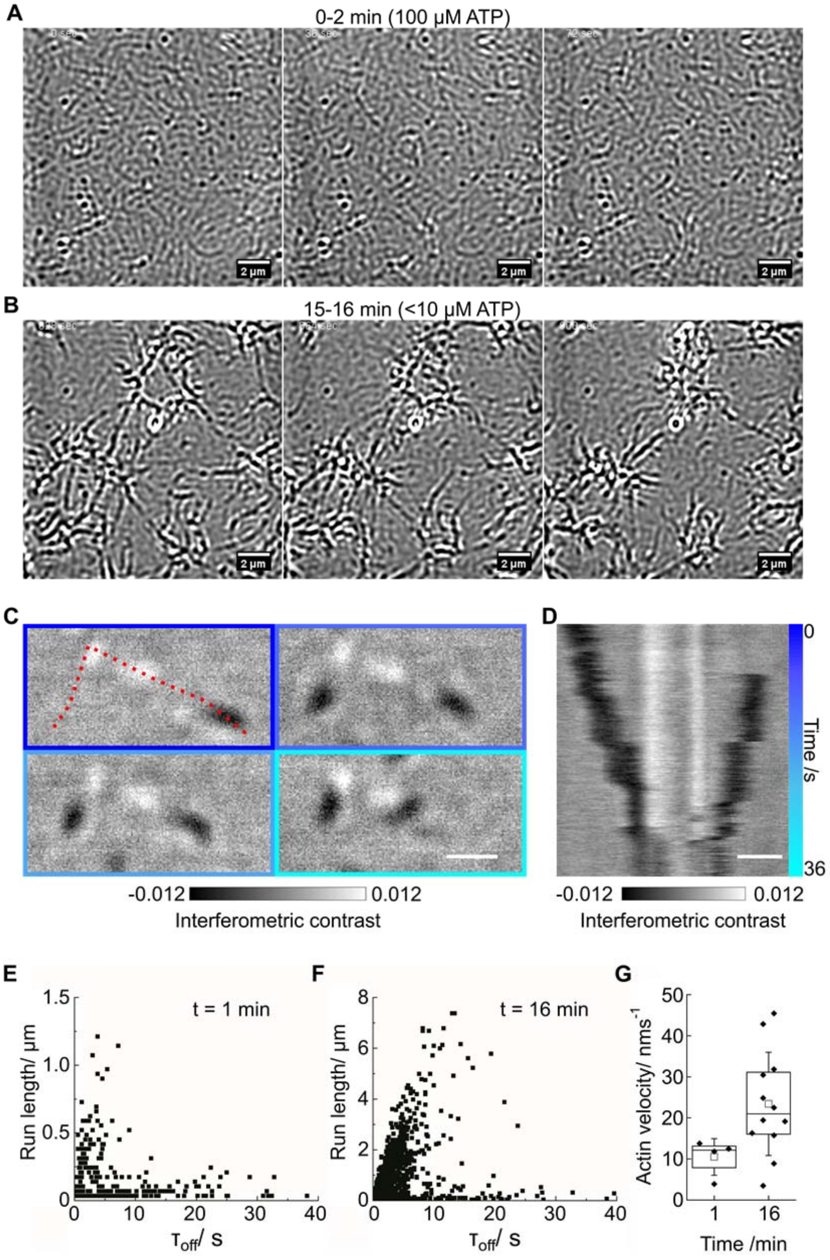

Figure S2, Myosin II filament dynamics: A, B) Image sequence depicting the dynamics of a membrane bound acto-myosin network at (**A**) the start of the experiment, in the remodeling state at 100 µM ATP and (**B**) after 16 min, when the network becomes contractile; images were acquired with the 445 nm laser iSCAT setup; scale bar: 2 µm. **C)** Image sequence showing the run of two myosin II filaments (black) on actin filaments; 635 nm laser iSCAT setup, scale bar: 1 µm. **D)** Corresponding kymograph obtained along the red lines in (C), black regions correspond to myosin II filaments that are moving along actin filaments; time color bar: 36 s, scale bar: 1 µm. **E)** Plot of run length vs binding time of myosin II filaments at t = 1 min (100 µM ATP) (Fig. 2E). **F)** Plot of run length vs binding time of myosin II filaments at t = 16 min (Fig. 2H). **G)** Boxplot depicting the velocity of actin filaments in single regions of the acto-myosin network indicating a very limited impact of actin filament displacement on the myosin II filament velocity measurements.

Figure S3

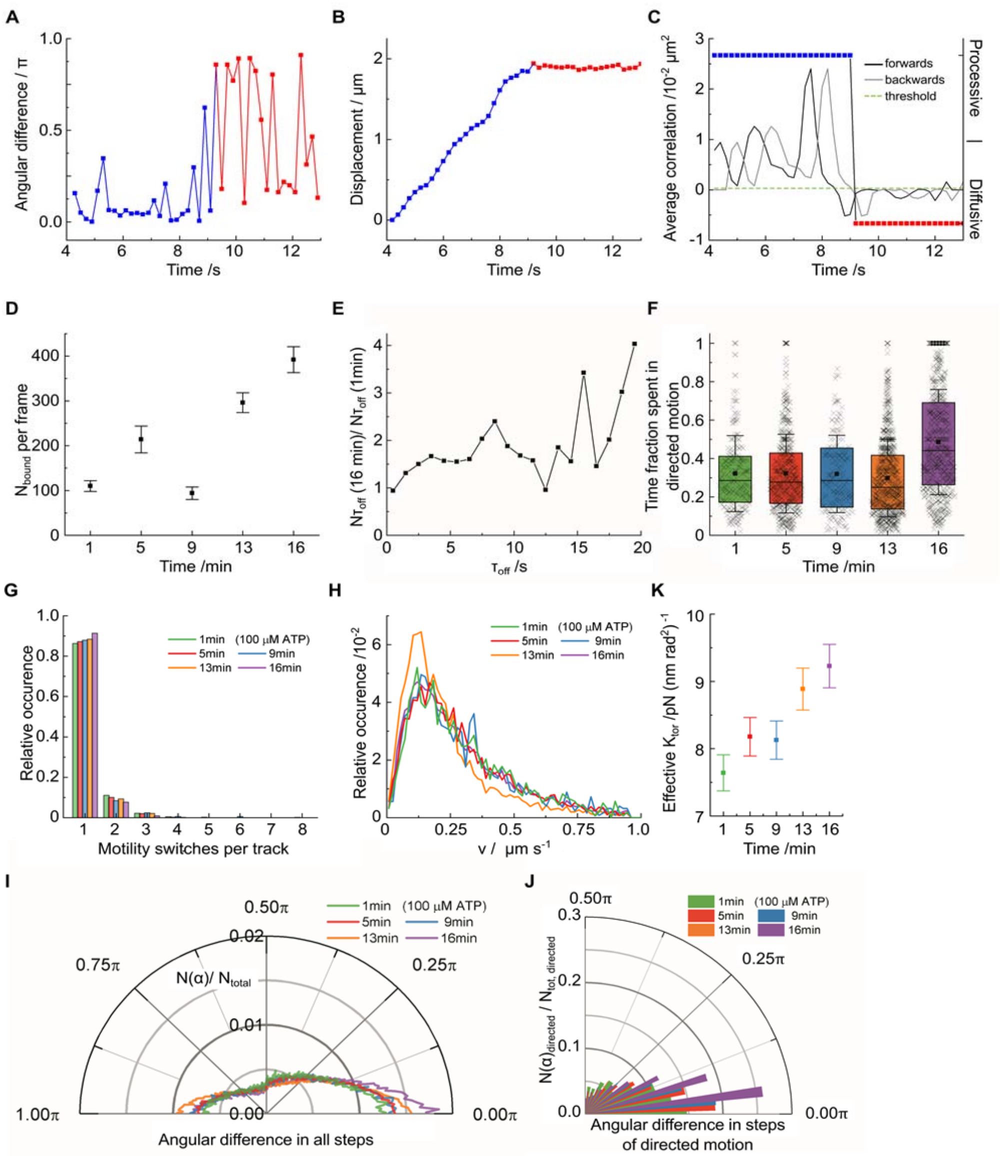

Figure S3, Myosin II filament single-particle tracking: A-C) Example of the routine to classify track segments of directed or random motion: based on the tracking data, the angular difference between particle orientation and direction of motion (**A**), the straight-line distance from the particle's origin (**B**) and the average correlation in displacements between adjacent timesteps (forward: $C_+ = \frac{\boldsymbol{dr}_{t+2\Delta t}\cdot \boldsymbol{dr}_{t+\Delta t} + \boldsymbol{dr}_{t+\Delta t}\cdot \boldsymbol{dr}_t}{2}$, backward: $C_- = \frac{\boldsymbol{dr}_{t-2\Delta t}\cdot \boldsymbol{dr}_{t-\Delta t} + \boldsymbol{dr}_{t-\Delta t}\cdot \boldsymbol{dr}_t}{2}$) were calculated (**C**) to reduce false positive detection; segments of correlation of displacement above the threshold (here 0.5 nm^2 as calculated from the average diffusivity of all myosin II filaments at t = 1 min) were classified as directed motion (blue dotted line), whereas segments below the threshold were deemed to be of random motion (red dotted line). **D)** Plot of the average (± sdv) number of automatically detected myosin II filaments per frame in the recordings at the indicated timepoints (N > 250 frames for each condition). **E)** Plot of the ratio of the numbers of detected myosin II filaments that exhibit each binding time at t=16 min (contractile state) and t = 1 min (remodeling state), indicating a 1.6-fold increase for binding times longer than 3 s; counts were normalized to the total number of detections at t = 1 min and t = 16 min. The strong fluctuations at times longer than 10s are due to the small numbers (< 10) of detected events. **F)** Box plot depicting the ratio of the duration of directed motion versus dwell time for each myosin II filament displaying directed motion at the indicated time points (N(1, 5, 9, 13, 16 min) = [221, 374, 158, 608, 640]). **G)** Histogram of the switches of each tracked myosin between directed and undirected motion at the indicated time points (sample numbers are

similar as in F); counts were divided by the total number of counts. **H)** Myosin II filament velocity distribution during directed motion based on the displacement between consecutive frames (Δt = 0.2 s) at the indicated time points of the experiment; counts were divided by the total number of counts. **I)** Radial plot depicting the angular difference α between myosin II filament orientation and its velocity vector for all detected myosin II filament steps at the indicated time points. **J)** Radial histogram plot depicting the ratio of steps at directed motion versus all detected steps for each angular difference α at indicated time points. **K)** Plot of the average (±std) effective torsional spring constant of myosin II filaments at the indicated time points; std estimation is based on the error of myosin orientation detection.

Table S1. Summary of the experimental conditions

Figure	[Actin]/ nM	[CP]/ nM	$L_{F\text{-actin}}$ /µm	[Myosin II]/ nM	$[ATP]_{initial}$/ µM	λ(laser)/ nm
1B	100	0	NA	0	0	445
1C	0	0	0	20	0	445
1E-H	125	0.75	3 ± 1.4	0	100	445
2, 3	300	0	7 ± 3.5	100	100	635
4 A, B	300	0	7 ± 3.5	100	100	635
S2A, B	250	0.5	2 ± 1.4	30	100	445

Table S1, experimental conditions: List of the experimental conditions used in the examples depicted in Figs. 1–4. The table contains the concentration of G-actin, capping protein (CP), myosin II proteins and ATP as well as the average length of actin filaments attached to SLBs, which was measured before the addition of myosin II. Sample size for filament length measurements: N = 505 (Fig.1E-H), N = 151 (Fig 2, 3, 4), N = 255 (Fig S2A, B).
