# Supplementary material for: Myosin II Filament Dynamics in Actin Networks Revealed with Interferometric Scattering Microscopy
Source: Biophys J. 2020 Mar 4;118(8):1946–57. doi: 10.1016/j.bpj.2020.02.025 (PMC7175421; doi:10.1016/j.bpj.2020.02.025)
Supplement: Document S2. Article plus Supporting Material [file mmc11.pdf]

Biophysical Journal
Article

Biophysical Society

Myosin II Filament Dynamics in Actin Networks Revealed with Interferometric Scattering Microscopy

Lewis S. Mosby,[1,2] Nikolas Hundt,[3] Gavin Young,[3] Adam Fineberg,[3] Marco Polin,[1,2] Satyajit Mayor,[4,5,*] Philipp Kukura,[3,*] and Darius V. Köster[1,6,*]

[1]Centre for Mechanochemical Cell Biology and [2]Physics Department, University of Warwick, Coventry, United Kingdom; [3]Physical and Theoretical Chemistry Laboratory, Department of Chemistry, University of Oxford, Oxford, United Kingdom; [4]National Centre for Biological Sciences, Tata Institute for Fundamental Research, Bangalore, India; [5]Institute for Stem Cell Biology and Regenerative Medicine, Bangalore, India; and [6]Division of Biomedical Sciences, Warwick Medical School, University of Warwick, Coventry, United Kingdom

ABSTRACT The plasma membrane and the underlying cytoskeletal cortex constitute active platforms for a variety of cellular processes. Recent work has shown that the remodeling acto-myosin network modifies local membrane organization, but the molecular details are only partly understood because of difficulties with experimentally accessing the relevant time and length scales. Here, we use interferometric scattering microscopy to investigate a minimal acto-myosin network linked to a supported lipid bilayer membrane. Using the magnitude of the interferometric contrast, which is proportional to molecular mass, and fast acquisition rates, we detect and image individual membrane-attached actin filaments diffusing within the acto-myosin network and follow individual myosin II filament dynamics. We quantify myosin II filament dwell times and processivity as functions of ATP concentration, providing experimental evidence for the predicted ensemble behavior of myosin head domains. Our results show how decreasing ATP concentrations lead to both increasing dwell times of individual myosin II filaments and a global change from a remodeling to a contractile state of the acto-myosin network.

SIGNIFICANCE Here, we show that interferometric scattering microscopy in combination with single-particle tracking enables label-free, high-contrast imaging of filament dynamics on surfaces while distinguishing different species based on their mass. These results significantly broaden the available tool kit and associated capabilities of researchers studying dynamics of biological machines at interfaces.

INTRODUCTION

The dynamics of the cell surface and many cellular processes depend on the interplay between the plasma membrane and the tightly associated dynamic actin cortex (1,2). Reconstituted minimal systems of membrane-bound acto-myosin networks are often employed to study physical principles controlling the dynamics of such active composites. Despite their relative simplicity, these systems can adopt a range of active states depending on ATP concentration, actin-to-myosin ratio, and actin filament length distribution and concentration (3,4). As a result, identifying the processes that lead from a remodeling, fluid-like acto-myosin network to a contractile, solid-like network has remained a considerable experimental challenge.

Most experimental and theoretical studies of myosin motor properties have relied on experiments with single-motor head domains. Recent advances in the theoretical understanding of myosin II filaments formed of multiple head domains indicate that small changes in the single-head duty ratio can lead to a switch from a nonprocessive state characterized by weak actin binding without continuous motion along actin filaments to a state of continuous actin binding, efficient motion along actin filaments, and force generation due to cooperative effects

Submitted October 9, 2019, and accepted for publication February 25, 2020.

*Correspondence: mayor@ncbs.res.in or philipp.kukura@chem.ox.ac.uk or d.koester@warwick.ac.uk

Lewis S. Mosby and Nikolas Hundt contributed equally to this work.

Nikolas Hundt's present address is Department of Cellular Physiology, Biomedical Center, Ludwig-Maximilians-Universität München, 82152 Planegg/Martinsried, Germany.

Editor: Michael Ostap.

https://doi.org/10.1016/j.bpj.2020.02.025

© 2020 Biophysical Society.

This is an open access article under the CC BY license (http://creativecommons.org/licenses/by/4.0/).

(5–8). To experimentally test the dynamics and properties of multiheaded myosin II filaments, it is necessary to visualize the network components with a subsecond time resolution over timescales of tens of minutes, which has been challenging to achieve with fluorescent probes because of photobleaching and phototoxicity.

Here, we employed interferometric scattering (iSCAT) microscopy (9,10), a label-free imaging technique that makes use of the interference between reflected and scattered light from nano-objects near an interface. The key advantages of light scattering over fluorescence detection in this context are the lack of an upper limit to the fluorescence emission rate and the absence of photobleaching and thus phototoxicity, enabling long observation times. We quantify microscopic quantities, such as actin filament mobility, and myosin filament dwell times and processivity while simultaneously monitoring mesoscopic phenomena, such as network flows and clustering. This approach allows us to link changes in the mechanochemical properties of myosin II filaments to transitions in the acto-myosin network, namely from the remodeling to the contractile state.

MATERIALS AND METHODS

Protein purification

Actin was purified from chicken breast following the protocol from Spudich and Watt (11) and kept on ice in monomeric form in G-buffer (2 mM Tris, 0.2 mM ATP, 0.5 mM tris(2-carboxyethyl)phosphine (TCEP)-HCl, 0.04% NaN_3, 0.1 mM $CaCl_2$ (pH 8.0)). Myosin II was purified from chicken breast following a modified protocol from Pollard (12) and kept in monomeric form in myo-buffer (500 mM KCl, 1 mM EDTA, 1 mM dithiothreitol, 10 mM HEPES (pH 7.0)). The day before experiments, functional myosin II proteins were separated from proteins containing dead head domains by a round of binding and unbinding to actin filaments at a 5:1 actin-to-myosin ratio (switch from no ATP to 3 mM ATP), followed by a spin at 60,000 rpm for 10 min at 4°C in a TLA100.3 rotor. The supernatant containing functional myosin II is dialyzed against myo-buffer overnight and used for experiments for up to 3 days.

To control the length of actin filaments, we titrated purified murine capping protein to the actin polymerization mix as described in (4). To link actin to the SLB, we used a construct containing 10× His domains followed by a linker (KCK) and the actin binding domain of ezrin (HKE) as described earlier (13).

Supported lipid bilayer and experimental chamber preparation

Glass coverslips (#1.5 borosilicate; Menzel, Germany) for SLB formation were cleaned with Hellmanex III (Hellma Analytics, Mühlheim, Germany) following the manufacturer's instructions, followed by thorough rinses with EtOH and MilliQ water, blow dried with N_2, and finally passed briefly over a Bunsen burner flame. For the experimental chamber, 0.2-mL PCR tubes (Tarsons Products, Kolkata, India) were cut to remove the lid and conical bottom part. The remaining ring was stuck to the cleaned glass using UV glue (NOA88; Norland Products, Cranbury, NJ) and 3 min of curing by intense UV light at 365 nm (PSD-UV8T; Novascan, Ames, IA). Freshly cleaned and assembled chambers were directly used for experiments.

Supported lipid bilayers (SLBs) containing 98% 1,2-dioleoyl-sn-glycero-3-phosphocholine (DOPC) and 2% 1,2-dioleoyl-sn-glycero-3-[(N-(5-amino-1-carboxypentyl)iminodiacetic acid)succinyl] (nickel salt) (DGS-NTA(Ni^{2+})) lipids were formed by fusion of small unilamellar vesicles as described previously (4). Prior to experiments with actin filaments, we formed SLBs in chambers filled with 100 μL KMEH (50 mM KCl, 2 mM $MgCl_2$, 1 mM EGTA, 20 mM HEPES (pH 7.2)). SLB formation was observed live using iSCAT microscopy to ensure that sufficient small unilamellar vesicles fused to form a uniform, continuous lipid bilayer (Video S1). Because the conditions and lipid composition used here were similar to our previous work, and based on the observation of the diffusive behavior of short actin filaments (see below), we assumed a fluid bilayer even though we did not test it directly by measuring the mobility of lipids or the lipid anchored HKE.

Formation of acto-myosin network

In a typical experiment, SLBs were formed, incubated with 10 nM HKE for 40 min, and washed three times with KMEH. The typical HKE density in our setup was estimated to be 3600–5700 HKE μm^{-2} ($\rho_{NiNTA} = 2\% * 1/\ 70\mathring{A}(A_{\text{lipid}}) = 28,571\ \mu m^{-2}$ (14), and if we assume that the decahistidine binds to five to eight NiNTA lipids, we obtain a protein density of 3600–5700 proteins per μm^2). Our decahistidine constructs and preparation protocols are as described in (15), which reports similar protein densities. During the incubation of HKE, actin filaments were polymerized. First $10\%_{\text{vol}}$ of 10× ME buffer (100 mM $MgCl_2$, 20 mM EGTA (pH 7.2)) was mixed with the G-actin and, optionally, with the capping protein stock and incubated for 2 min to replace G-actin-bound Ca^{2+} ions with Mg^{2+}. Addition of 2× KMEH buffer supplemented with 2 mM Mg-ATP induced actin filament polymerization at a final G-actin concentration of 5 μM. After 20–30 min of incubation, the desired amount of actin filaments was added to the SLBs using blunt-cut 200-μL pipette tips. An incubation of 30 min allowed the actin filament layer to reach an equilibrium state of binding to the SLB. We prepared myosin filaments by diluting the stock ($C_{\text{myoII}} = 4\ \mu M$; 500 mM KCl, 1 mM EDTA, 1 mM dithiothreitol, 10 mM HEPES (pH 7.0)) 10 times with MilliQ water to drop the KCl concentration to 50 mM and incubating for 5 min to ensure myosin filament formation. Myosin filaments were added to the actin network by replacing 1/10 of the sample buffer with the myosin II filament solution and supplementing with Mg-ATP (100 mM) at 0.1 mM final concentration. To summarize, the final buffer composition was 50 mM KCl, 2 mM $MgCl_2$, 1 mM EGTA, 20 mM HEPES, 0.1 mM ATP (pH 7.2), containing actin filaments (corresponding to $C_{\text{G-actin}} = 100$–300 nM) and myosin II filaments ($C_{\text{myoII}} = 0$–100 nM). It was important to keep the pH at 7.2 because changes in pH would affect motor activity. Afterward, the dynamics of the acto-myosin system were observed for up to 60 min. The system usually showed a remodeling behavior for the first 10–15 min before contraction and aster formation (because of ATP concentrations dropping below 10 μM as estimated from the activity of myosin II and earlier reports (16)). Once the system reached a static, jammed state and no remaining myosin activity was observed, the system could be reset into a remodeling state by addition of Mg-ATP (100 mM) to a final concentration of 0.1 mM (Video S6). Each step of this procedure was performed on the microscope, which allowed us to check the state continuously. The open-chamber design allowed the addition of each component from the top without induction of flows that would perturb the actin network. Evaporation was below 5% during a period of 60 min, so salt and protein concentrations can be considered constant over the time course of a typical experiment (Fig. S1 *A*). All experiments were performed at room temperature (22°C).

Details about the actin, myosin, and ATP concentrations used in each experiment can be found in Table S1.

iSCAT microscopes

The principle of iSCAT microscopy is based on the interference of light reflected from the glass substrate with the light that is scattered from the particle of interest, and the measured intensity at the detector is given by

$$I_{iSCAT} = |E_i|^2\{r^2 + s^2 + 2r|s|\cos\varphi\}, \quad (1)$$

where $|E_i|^2 r^2$ describes the light intensity reflected by the glass-water interface (r ~0.065), $|E_i|^2 s^2$ is the pure scattering contribution (negligible for very weak scatterers with an interferometric contrast of <30%), and $|E_i|^2 2r|s|\cos\varphi$ denotes the interference between scattered and reflected light, with φ being the phase difference between the two (17). iSCAT experiments were performed on two different home-built setups similar to those detailed in (18). Briefly, a weakly focused laser beam was scanned across the sample over an area of 24 × 24 μm^2 (445-nm laser) or 32.6 × 32.6 μm^2 (635-nm laser). The light reflected from the glass-water interface together with the scattered light from the sample was imaged onto a CMOS camera (445-nm laser: Point Gray GS3-U3-23S6M-C; Flir, Canada; 635-nm laser: MV-D1024-160-CL-8; Photonfocus, Switzerland). The cameras were controlled using home-written LabVIEW software. The setup with the 445-nm laser had a 3.5-mm partially reflective mirror placed in the reimaged back focal plane of the objective for enhanced scattering contrast as described in (18). The illumination intensity on the sample (445-nm laser: 250 W/cm^2; 635-nm laser: 1.9 kW/cm^2) was set to nearly saturate the camera with the returning light. The pixel sizes were 23.4 nm/pixel (445-nm laser) and 31.8 nm/pixel (635-nm laser).

Image processing

The videos were recorded at 50 fps (445-nm laser) and 25 fps (635-nm laser) and preaveraged by a factor of 5 to reduce noise. Nonsample-specific illumination inhomogeneities, fixed-pattern noise, and constant background were removed from the raw images by dividing each of them with a flat field image that contained only these features. The flat field image was computed by recording 2000 frames of the sample while moving the stage. For each pixel, a temporal median was calculated, resulting in a flat field image that only contained static features.

Median filtering

Videos were median filtered using MATLAB (The MathWorks, Natick, MA). For each image sequence, the median is computed for each pixel and subtracted from the original image sequence, and the median-filtered image sequence as well as the computed median filter are saved.

Actin filament tracking

Actin filaments that became visible after median filtering and that did not cross other actin filaments for at least 1000 frames were tracked using ImageJ (http://imagej.nih.gov) and the plugin JFilament (19,20). The obtained tracking traces were analyzed using MATLAB to compute the position of the center of mass and the filament orientation for each time point and to generate plots of the center of mass total mean-square displacement (MSD) as well as the parallel and perpendicular components of the MSD with respect to the filament orientation.

Actin filament length measurements

Image stacks of actin filaments landing on the HKE-decorated SLBs were taken at 10 Hz immediately after addition of actin filaments to the sample. Theses image stacks were split in segments of 10 s, and the median of each segment was subtracted from its last frame to visualize freshly landed, isolated actin filaments. The images were then converted from the 32-bit interferometric contrast values to 8-bit (by the formula $f(x) = 1000 \times (-x) + 1000$), bandpass filtered (low pass: 3 pixel, high pass: 20 pixel), and analyzed with the ImageJ plugin NeuronJ (21).

Actin filament layer thickness measurements

The maximal interferometric contrast values of randomly drawn line scans across SLB-bound actin networks before the addition of myosin were taken and divided by the average interferometric contrast value of a single actin filament (see Fig. 1 *D*) to obtain an estimate of the actin network thickness as a number of actin filaments. Notably, the obtained maximal thickness of six actin filaments would amount to a network height of ~6 × 8 nm = 48 nm, which lies within the working distance of the objective used.

Myosin II filament length measurements

Line scans along the long axis of single myosin filaments were taken, and the distance between the half-maximal points at both ends was taken as the length of the myosin II filament.

Myosin binding dynamics

Imaging with an effective frame rate of 5–10 Hz for several minutes was sufficient to capture a broad range of myosin filament dynamics with high accuracy. To remove any signal originating from static structures, such as immobile actin filaments or small impurities in the SLB, the image sequences were median filtered (22). In a second step, a maximal projection of the time series was used to visualize the tracks taken by myosin II filaments during the experiment. Lines following these tracks were then used to compute kymographs depicting the myosin II filament binding times and their motion along actin filaments (kymograph tool in ImageJ, line width 3). Dwell time, run length, and velocity distributions were plotted and further analyzed using OriginPro 2018 (OriginLab, Northampton, MA) and MATLAB. Best-fitting functions for the myosin II filament dwell times were selected using MEMLET, which estimates the maximum likelihood of a fitting function to describe a data point distribution (23).

Detection and tracking

Image analysis and single-particle tracking was carried out using ImageJ and Python code developed for this work (detailed description in (24), www.github.com/cmcb-warwick/myoSPT). Briefly, individual myosin filament detection was implemented in the Python programming language using the Sep package (based on the algorithms of Source Extractor) (25–27), which generates the position, spatial extent, and orientation of the ellipsoidal myosin II filaments for each frame. Based on the amplitude of spatial displacement, orientation changes and the detected particle area tracks were generated and classified into regions of random or directed motion.

RESULTS

Detection and characterization of actin and myosin filaments

The critical step to using label-free imaging for acto-myosin dynamics on a membrane is the detection and distinction of actin and myosin II filaments. Because of the absence of crowding factors or excess proteins in our experiments, the actin and myosin II filaments were the principal sources of light scattering after subtraction of background signatures originating from cover glass roughness and leading to interference with the light reflected from the cover glass surface, referred to in the following as interferometric contrast with values <0 (Figs. 1 *A* and S1 *B*; (22,28)). Myosin II and actin filaments landing on bare glass slides exhibited

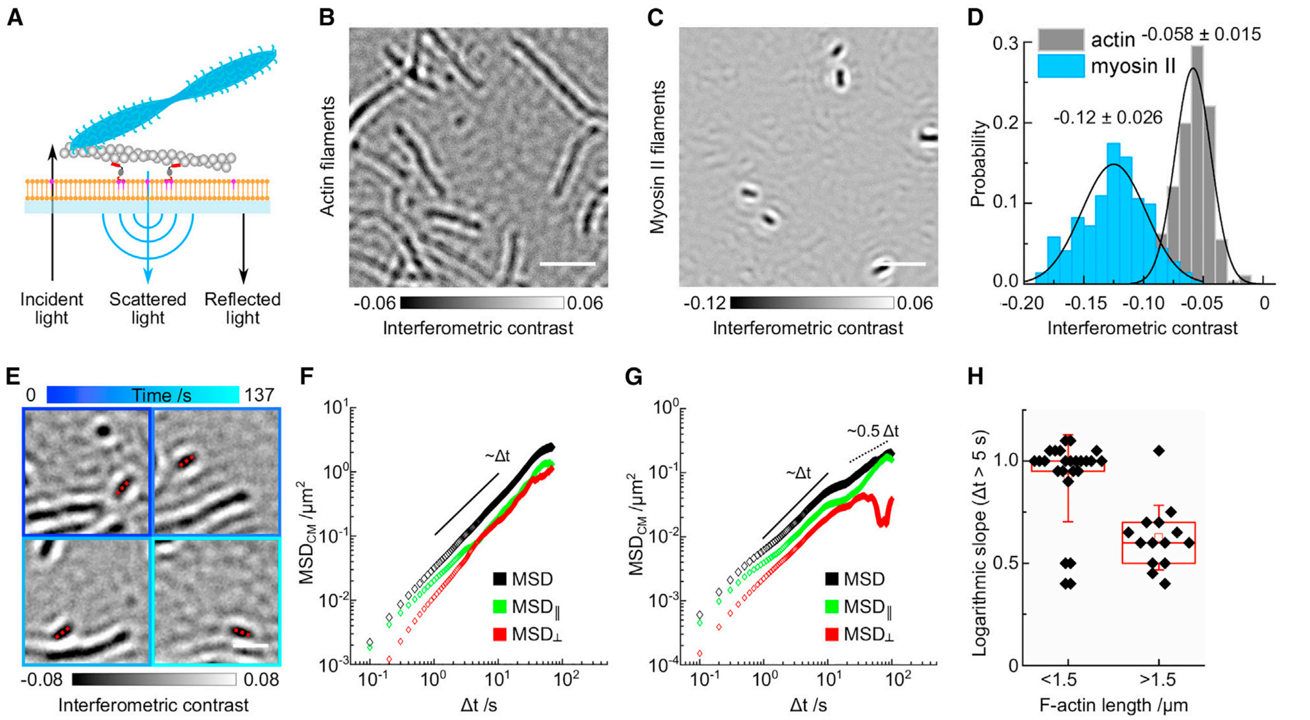

FIGURE 1 Experimental setup. (*A*) Shown is a diagram of the in vitro system consisting of a supported lipid bilayer (*orange*), actin-membrane linker protein decahistidine-KCK-ezrin actin binding domain (HKE; *dark gray* and *red*), actin filaments (*gray*), and muscle myosin II filaments (*blue*); arrows indicate principle of iSCAT microscopy. (*B* and *C*) Shown are example images of actin filaments (*B*) and myosin II filaments (*C*), both recorded with a 445-nm laser iSCAT system. Scale bars, 2 μm. (*D*) Shown is a histogram depicting the interferometric contrast distribution along actin filaments (*gray*, $N_{measure} = 562$ measurements along $N_{fil} = 12$ filaments) and myosin II filaments (*blue*, $N_{measure} = 303$ measurements along $N_{fil} = 14$ filaments); solid lines depict fits of a normal distribution with mean $\pm$ standard deviation noted above them. (*E*) Shown is an example of tracking a single actin filament (*red dashed line*, $l = 0.4$ μm) inside an actin network imaged over 137 s (from *dark blue* to *cyan*: 0–137 s; Video S3). Scale bars, 1 μm. (*F*) Shown is the corresponding MSD of the filament's center of mass (*black*) and the components parallel (*green*) and perpendicular (*red*) to the actin filament orientation. (*G*) Shown is the MSD of the center of mass of a long actin filament ($l = 4$ μm, Video S4) (*black*) and its components parallel (*green*) and perpendicular (*red*) to the actin filament orientation, indicating confinement at timescales >10 s. (*H*) Shown is a box plot comparing the diffusive behavior (characterized by logarithmic slopes of MSD plots) of short (<1.5 μm, N = 25) and long (>1.5 μm, N = 14) actin filaments for $\Delta t = 5$ - 10 s indicating confined diffusion for filaments longer than 1.5 μm due to the surrounding actin meshwork, middle line: median, square: mean, whiskers: standard deviation. To see this figure in color, go online.

interferometric contrasts of -0.120 ± 0.026 and -0.058 ± 0.015, respectively, using an iSCAT setup equipped with a 445-nm laser (Fig. 1, *B–D*). The interferometric contrast values were smaller using a 635-nm laser setup, with values of -0.017 ± 0.006 for myosin II filaments and -0.002 ± 0.001 for actin filaments, respectively (Fig. S1 *C*). The contrast ratio with the 635-nm laser setup ($0.017/0.002 = 8.5$) was larger than with the 445-nm laser setup and reflected the mass ratio of actin to myosin II filaments per unit length (within a diffraction-limited spot of 210 ± 10 nm: $m_{myoII}/m_{F-actin} = [42 \text{ dimers} \times 520 \text{ kDa}]/[60 \textit{ subunits} \times 42 \text{ kDa}] = 8.7$) (29–31) and the linear scaling of the interferometric signal with molecular mass (28). The weaker contrast difference measured with the 445-nm laser ($0.12/0.06 = 2$) was likely due to a non-negligible contribution from direct scattering by the large myosin II filaments (the $|s|^2$ term in Eq. 1), leading to a reduced interferometric contrast at high molecular masses. In our buffer conditions containing 50 mM KCl, myosin II filaments exhibited an average length of 520 ± 130 nm (N = 269) (Fig. S1 *D*), as reported earlier (3). The clear difference in interferometric contrast with the 635-nm laser setup and the characteristic, uniform shape of myosin II filaments provided a solid basis to distinguish myosin II filaments reliably from actin filaments, whereas the 445-nm laser setup was mainly used to track actin filament dynamics.

Next, we characterized the formation of actin networks on SLBs. After directly observing successful SLB formation (Video S1), incubation with an actin-membrane linking protein (decahistidine-KCK-ezrin actin binding domain, HKE; after median subtraction, HKE-decorated SLBs displayed interferometric contrast fluctuations of ± 0.005 on the 445-nm laser setup), and addition of actin filaments, regions exhibiting consecutive deposition of multiple actin filaments within a diffraction-limited spot displayed a stepwise increase in the interferometric signal (Fig. S1, *E* and *F*; Video S2). By dividing local contrast measurements by the average value for a single actin filament (-0.058 ± 0.015), we estimated that, under the conditions used here ([G-actin] = 100–350 nM), the actin layers were one to

six filaments thick, with two filaments per diffraction-limited spot being most frequent (Fig. S1 *G*). This is entirely consistent with previous results using fluorescently labeled actin filaments (4).

It was important to check for the fluidity of the supported lipid bilayers to ensure the lateral mobility of the membrane-actin linker HKE and, hence, the remodeling of the membrane-tethered actin network. Because HKE could not be visualized directly using iSCAT due to its low molecular weight (15 kDa) (13), we assessed the mobility of individual short actin filaments as a proxy. Offline median subtraction (median of 2-min image sequences) removed quasistatic actin network components and revealed the mobile filament fraction (Fig. 1 *E*; Video S3). Tracking of individual filaments and analysis of their MSD indicated a fluid lipid bilayer with free diffusive behavior for most actin filaments shorter than 1.5 μm on timescales of 5 s and longer, usually reaching MSD values of >0.1 μm^2 (Figs. 1, *E*, *F*, and *H* and S1, *H* and *J*). Longer filaments also displayed diffusive behavior on short timescales but were more confined with lower MSD values (in the range of 0.1 μm^2 at $\Delta t = 5$ s) characterized by lower slopes of the MSD (Figs. 1, *G* and *H* and S1, *I* and *K*; Video S4). Two major effects are likely to influence actin filament mobility here: the number of molecules tethering the actin filament to the membrane and steric effects by the surrounding actin network. Considering that longer actin filaments will have a higher number of HKE molecules tethering them to the bilayer, one can assume that the actin filament motility would decrease as a function of actin filament length, as was discussed in the case of microtubules in (32). One can assume that the number of HKE molecules under an actin filament would be ~30 per μm (0.005 μm $\times$ 5700 HKE μm^{-2} = 28.5 HKE μm^{-1}). The presence of a heterogeneous meshwork resulting in different confinements for actin filaments depending on their local environment could give rise to tracks showing non-Gaussian diffusion as described in (33) and as we observe (Fig. S1, *J* and *K*). However, a detailed analysis of whether actin filament diffusion could be used to reveal the actin network mesh size distribution or how actin mobility depends on actin filament length would be beyond the scope of this study.

Binding dynamics of myosin II filaments to actin

We then characterized myosin II filament binding to membrane-bound actin networks at 100 μM ATP (t = 1 min), which fueled continuous network remodeling for several minutes until it became contractile at about t = 16 min because of low ATP levels (Fig. S2, *A* and *B*; Video S5). Previously, we analyzed this transition, e.g., by calculating the spatial density correlation of actin, which changed clearly at the onset of the contractile state because of the clustering of actin (4). Given that we observed in this work similar network dynamics and timescales of the remodeling and contractile states, we did not perform a similar analysis but wanted to understand whether changes in myosin II filament dynamics could be associated to changing network dynamics. We followed myosin II filament dynamics using a 635-nm laser iSCAT setup over a time window of >16 min by recording multiple sequences of 2-min videos (3000 frames at 25 Hz) due to computer-hardware-related limitations for rapid data storage. After averaging over five frames to reduce noise levels, we obtained an effective frame rate of 5 Hz, which was 10–20 times faster compared with earlier studies using fluorescence light microscopy limited by phototoxicity (4,34). As described previously (4,16,35), we defined the onset of network contractility by the myosin-induced formation of actin clusters that continued to merge into larger structures until the system reached a jammed or static state without any visible actin or myosin motion. By contrast, the remodeling state was characterized by local, transient changes of the actin network without inducing any large-scale contractile flows. Changes in network contractility were due to changes in the available ATP, and addition of fresh ATP could reverse the strong binding of myosin II filaments (Video S6).

By creating kymographs along the tracks of myosin II filaments (Fig. S2, *C* and *D*; Video S7), we found that the distribution of myosin II dwell times on actin filaments followed a double exponential decay function. At t = 1 min, the computed time constants were τ_{off1} = 1.23 s ([1.12–1.36 s]$_{95\%}$) and τ_{off2} = 12.6 s ([11.3–14 s]$_{95\%}$), with 66–71% of the events being described by τ_{off1} (Fig. 2 *A*). At t = 16 min, the myosin II filament dwell time constants were τ_{off1} = 3.7 s ([3.1–4.2 s]$_{95\%}$) and τ_{off2} = 11.9 s ([9.4–15.5 s]$_{95\%}$), with 63–87% of the events being described by τ_{off1} (Fig. 2 *B*).

We went on to analyze the motion of myosin II filaments. At t = 1 min, the mobile myosin II filaments exhibited a velocity distribution that can be described by a sum of two Gaussians, with >60% of myosin II filaments traveling at $v_{myoII,1}$ = 0.04 ($\pm$0.03) μm s^{-1} and the remaining at $v_{myoII,2}$ = 0.20 $\pm$ 0.13 μm s^{-1} (Fig. 2 *C*). The corresponding run length distribution decays exponentially, with a characteristic run length of λ = 0.23 ($\pm$0.03) μm (Fig. 2 *D*). Plotting run length versus dwell time (i.e., total time of attachment during which the run happened) shows a moderate positive correlation (Pearson coefficient 0.38, *p*-value: 7 $\times$ 10^{-10}) for dwell times <3 s and a weak negative correlation (Pearson coefficient −0.17, *p*-value: 2 $\times$ 10^{-2}) for longer binding times (Figs. 2 *E* and S2 E). This implies that the population corresponding to short dwell times is mobile, whereas the population with long dwell times exhibits slow, reduced motion and eventually becomes immobilized.

At t = 16 min, myosin II filaments traveled with an average velocity of v_{myoII} = 0.46 $\pm$ 0.20 μm s^{-1} (Fig. 2 *F*, single Gaussian distribution), and the characteristic run length was λ = 1.03 $\pm$ 0.05 μm (Fig. 2 *G*). The correlation between run length and dwell time is stronger compared

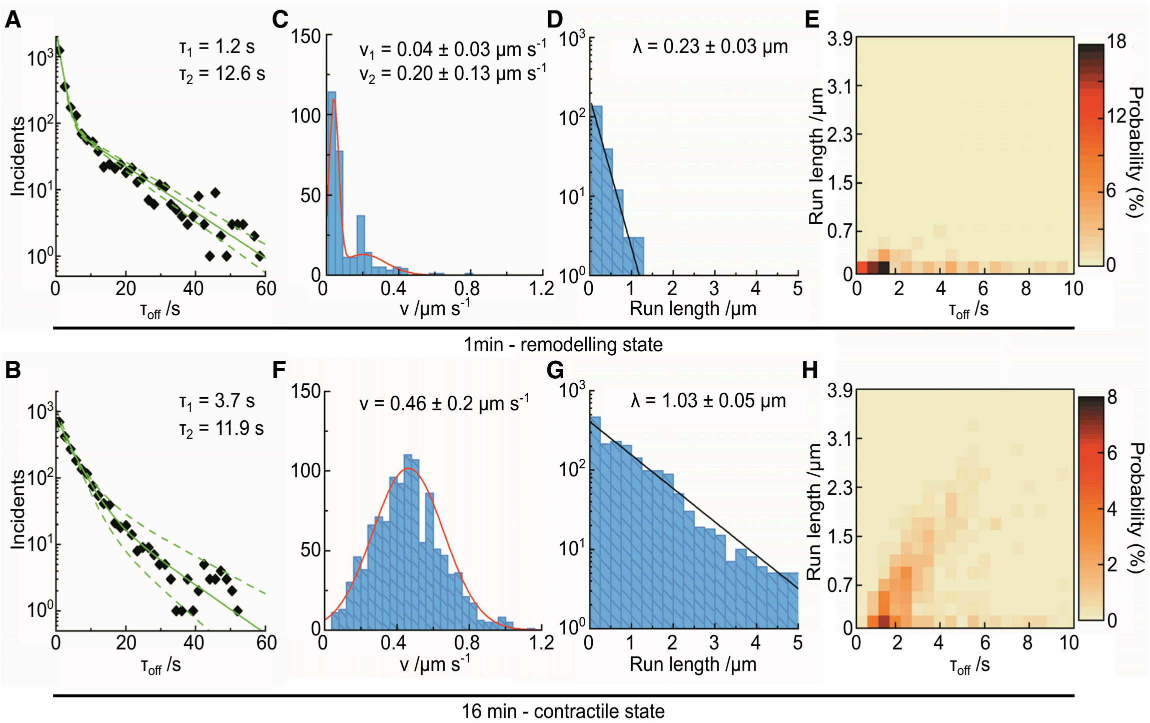

FIGURE 2 Myosin II filament dynamics in the acto-myosin network at different ATP concentrations. (*A* and *B*) Shown is a histogram of myosin II filament dwell times on actin at t = 1 min (100 μM ATP) (N = 6400) (*A*) and at t = 16 min (N = 8000) (*B*); each diamond represents a five frame = 1 s bin. (*C–E*) Shown are histograms of the myosin II filament velocities (*C*), run lengths (*D*), and frequency plot of run length versus dwell time (*E*) extracted from a subset of the myosin II filament kymographs at t = 1 min (N = 432). (*F–H*) Shown are histograms of the myosin II filament velocities (*F*), run lengths (*G*), and frequency plot of run length versus dwell time (*H*) extracted from a subset of the myosin II filament kymographs at t = 16 min (N = 1133). Best fits for the distributions were computed with the MEMLET fitting routine: (*A* and *B*) double exponential decay (*solid green line*, *dashed lines* indicate 95% confidence level), (*C*) double Gaussian (*red line*), (*F*) single Gaussian (*red line*), and (*D* and *G*) single exponential decay (*black line*). Data displayed in (*E*) and (*H*) are displayed in the range of 0–10 s to highlight this dwell time regime. To see this figure in color, go online.

with t = 1 min, with most myosin II filaments exhibiting persistent motion for up to 6 s (Pearson coefficient 0.57, p-value: 1×10^{-16}) and a loss of this correlation at longer dwell times (Pearson coefficient −0.18, p-value: 3×10^{-3}) (Figs. 2 *H* and S2 *F*).

To assess whether the measured velocities were a combination of myosin and actin network motion, we applied a 50-frame median filter (corresponding to 10 s) filtering out the fast-moving myosin II filaments and highlighting actin filament motion. The largest actin network displacements that we could observe occurred only locally and sporadically and did not exceed 50 nm s^{-1} in the contractile state at t = 16 min and 15 nm s^{-1} in the remodeling state at t = 1 min (Fig. S2 *G*). These observations agree with earlier studies (4,36) and underline that myosin II filament velocities result directly from motor activity on actin filaments. A comparison of the forces required to drag actin filaments and the attached actin-membrane binding proteins along the supported lipid bilayer with the drag forces experienced by myosin filaments moving through an aqueous buffer also suggests that myosin activity mainly propels myosin II filaments and is not considerably reduced by slippage of the membrane-tethered actin filaments (see Supporting Material) (32).

Myosin II filament tracking reveals links between binding mode, orientation, and mobility

Even though the use of kymographs to analyze binding dynamics and the mobility of motor proteins is well established, it bears some drawbacks, particularly when studying dynamics in complex remodeling networks: Kymographs only work well along static tracks, making it difficult to capture movements along actin filaments that change position over time; in addition, short binding events outside the kymograph lines would not be detected, or if captured, the reduced dimension due to the line scan would make it difficult to distinguish from noise. The characteristic shape and signal of myosin II filaments obtained with iSCAT microscopy, however, allowed us to develop an automated single-particle tracking algorithm to analyze the dynamics and orientation of individual myosin II filaments in a more detailed way than possible with kymographs. By analyzing the intensity distribution of pixels belonging to each myosin II filament, we could extract the particle location and orientation for each time point. Tracks were generated based on criteria including the particle size and the maximal displacement between frames (Fig. 3, *A* and *B*; Video S8) (24). Given the chosen intensity and area thresholds, detection

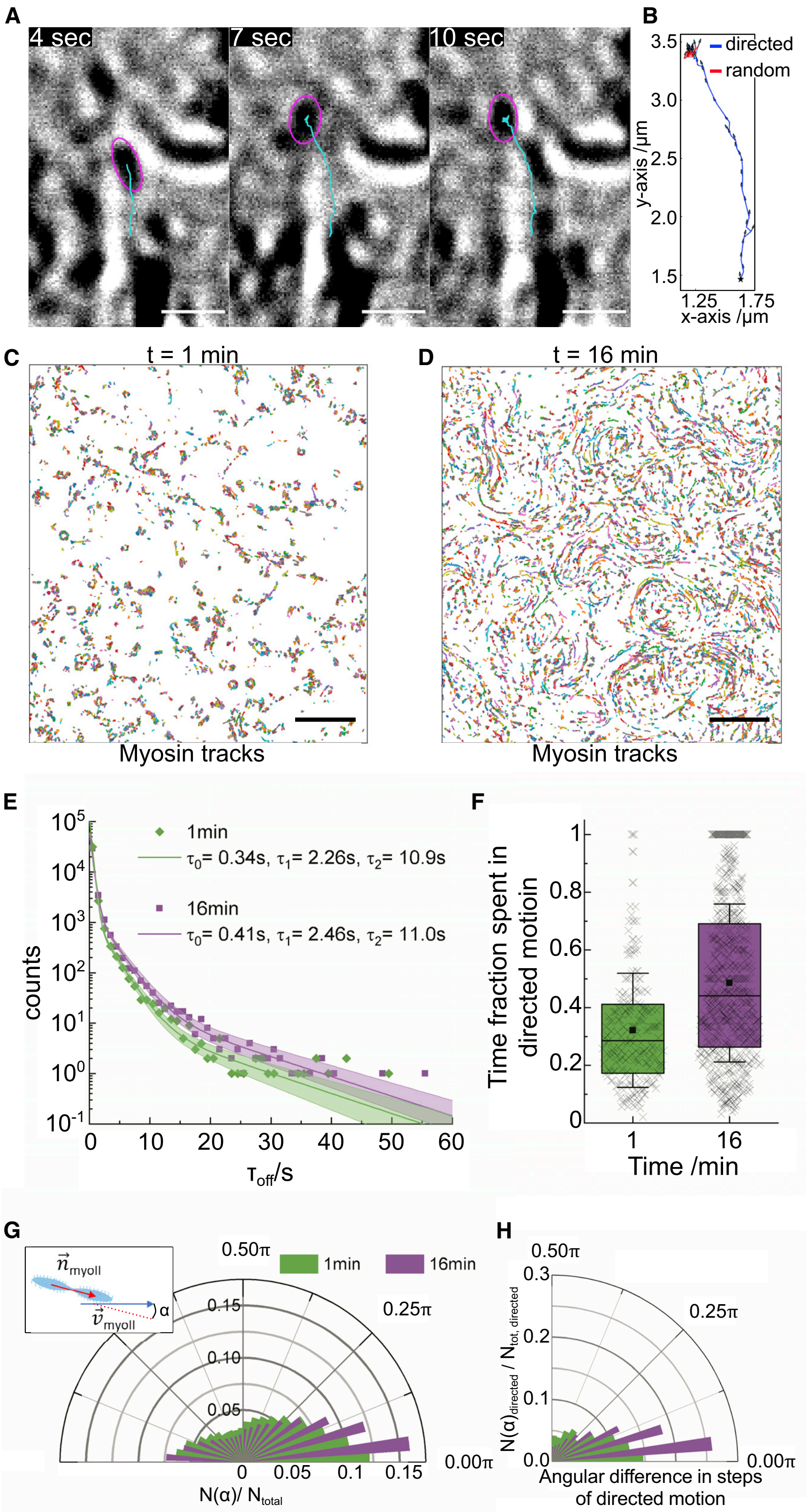

FIGURE 3 Single-particle tracking of myosin II filaments provides further insights into the change of myosin dynamics with decreasing ATP concentration. (*A*) Shown is an example image sequence showing the tracking of a myosin II filament (*purple circle* locates the current detection, *cyan line* depicts the particle's traces). Scale bars, 1 μm. (*B*) Corresponding track with segmentation into segments of directed (*blue*) and random (*red*) motion. (*C* and *D*) Images depicting all tracked myosin II filaments (in alternating *colors* to distinguish individual tracks) at (*C*) t = 1 min (100 μM ATP) (N = 14,016) and (*D*) t = 16 min (N = 15,647). Scale bars, 5 μm. (*E*) Shown is a corresponding histogram of myosin II filament dwell times on actin (t = 1 min: *green*; t = 16 min: *purple*); best fits for the distributions were computed with the MEMLET fitting routine: triple exponential decay with $t_{min} = 0.3$ s (*solid line*); boundaries depict 95% confidence level. (*F*) Shown is a box plot depicting the ratio of time of directed motion versus dwell time for myosin II filaments displaying directed motion ($N_{1\ min} = 221$; $N_{16\ min} = 640$). (*G*) Shown is a radial histogram depicting the angular difference α between myosin II filament orientation and its velocity vector for all detected myosin II filament steps. (*H*) Shown is a radial histogram plot depicting the ratio of steps in directed motion versus all detected steps for each angular difference α, indicating that there is a clear increase of filaments that are aligned along their axis of propagation in the contractile state. To see this figure in color, go online.

of unbound myosin II filaments moving by chance near the glass surface was very unlikely because it would move out of a 200-nm radius within less than 10 ms, which was significantly shorter than our image integration time (see Supporting Material). By quantifying the diffusivity of myosin II filaments exhibiting random motion calculated using

averages of the MSD over all myosin II filaments at t = 1 min, individual myosin II filament tracks could be divided into segments of random and directed motion (Figs. 3 *B* and S3, *A–C*). We used the average MSD data at t = 1 min (100 μM ATP) to calculate a threshold for identifying directed motion to classify individual tracks. The random motion or weak-binding state was characterized by an increased rate of large angular fluctuations and random displacements that resulted in linear time evolution of the MSD, resembling a diffusive particle.

This automated detection method provided a more comprehensive picture of myosin II filament dynamics in the remodeling state at t = 1 min and in the contractile state at t = 16 min (Fig. 3, *C* and *D*). Moreover, it increased the sensitivity for short binding times compared with the kymograph method. This higher detection sensitivity was reflected in the number of myosin II filaments identified in each frame, which fluctuated between 94 ± 14 and 214 ± 30 detections between t = 1 min and t = 9 min and rose to 392 ± 29 detections per frame at t = 16 min (Fig. S3 *D*). At t = 1 min (100 μM ATP), we found characteristic binding times of τ_{off0} = 0.34 s ([0.33–0.35 s]$_{95\%}$), τ_{off1} = 2.26 s ([2.13–2.41 s]$_{95\%}$), and τ_{off2} = 10.9 s ([9–12.8 s]$_{95\%}$) and at t = 16 min τ_{off0} = 0.41 s ([0.39–0.43 s]$_{95\%}$), τ_{off1} = 2.46 s ([2.33–2.63 s]$_{95\%}$), and τ_{off2} = 11 s ([10–12.5 s]$_{95\%}$) (Fig. 3 *E*). Interestingly, the characteristic times only increased slightly at t = 16 min, but significantly; performing a nonparametric test (Kolmogorov-Smirnov) highlighted that the t = 16 min dwell time distribution was shifted toward longer dwell times as compared with the t = 1 min distribution with $p = 0.04$ for dwell times >2 s. The number of events with binding times longer than 3 s increased >1.6-fold compared with the remodeling state (Fig. S3 *E*). The number of long-duration binding events in the contractile state could even be an underestimate because only binding-unbinding cycles occurring within the recording time were considered, discarding myosin II filaments that were bound for the entire length of the video or that were traveling into clusters or out of the field of view.

The fraction of myosin II filaments moving in a directed manner, however, increased nearly 3-fold ($N_{directed}/N$ = 221/35,644 at t = 1 min; $N_{directed}/N$ = 640/35,358 at t = 16 min). Concomitantly, the fraction of time each myosin II filament performed directed motion increased from 0.3 ± 0.2 at t = 1 min to 0.5 ± 0.3 at t = 16 min (Figs. 3 *F* and S3 *F*) of the total bound time, whereas the average number of switches between the two states of motion changed only from 1.1 ± 0.3 (N = 221) to 1.2 ± 0.5 (N = 640), suggesting that the additional time of directed motion at t = 16 min is spent in longer, continuous runs and not split up in multiple short sequences of directed and undirected motion (Fig. S3 *G*). This analysis is limited to runs lasting ≥10 frames (2 s) (24), and experiments at higher frame rates would be necessary to reveal dynamics at shorter timescales.

These results indicate that the contractile state is driven by both an increased total number of myosin II filaments showing directed motion and a higher persistence of each myosin II filament. The observed changes of myosin II filament run length and dwell time are most likely due to changes in ATP concentration and not due to changes in the actin filament length because the characteristic actin filament length at the onset of the experiments was 7 ± 3.5 μm and, hence, exceeded the run lengths we measured for myosin II filaments at t = 16 min (Fig. 2 *G*). We analyzed the myosin II filament displacement per frame (0.2 s) of all tracks during periods of directed motion to assess whether the displacement per myosin head stroke or the number of myosin head strokes per second changed during the course of the experiment but did not find any detectable changes over the course of the experiment (Fig. S3 *H*). This implies that the velocity distribution of myosin II filaments at directed motion remained constant over the range of 10–100 μM ATP and indicates that the difference in velocities measured with the kymograph method (Fig. 2, *C* and *F*) might have been due to the comparison of tracks comprising intervals of both directed and random motion.

By computing the angle α between myosin II filament orientation and its velocity direction for each myosin II filament step, we found that α varied significantly at ±45° full width at half maximum (FWHM) at t = 1 min, whereas it narrowed with increasing time to ±25° FWHM at t = 16 min (Figs. 3 *G* and S3 *I*). Importantly, at t = 16 min, the majority of myosin II filaments moving in a directed fashion is aligned with their direction of propagation (Figs. 3 *H* and S3 *J*). This improved alignment with the actin tracks at t = 16 min could be due to higher numbers of myosin head domains binding to actin filaments, which would support long-range transport and buildup of persistent material flows. As an approximation, we computed the effective rotational flexibility of myosin II filaments if the observed angular fluctuations (α) would solely be driven by thermal energy (k_BT) using the relation $k_BT = \langle\Delta\alpha^2\rangle K_{tor}$, with K_{tor} being the effective torsional spring constant of the myosin II filament around its attachment point, and found that K_{tor} increased from 7.5 to 9.2 pN nm^{-1} rad^{-2} during the time of the experiment (Fig. S3 *K*). This is ~3 times below the reported value for an individual myosin II head domain of 23 pN nm^{-1} rad^{-2} (37–39), which might be due to several factors: 1) experiments performed in solution in contrast to the previous studies using myosin II head domains adsorbed and fixed on EM grids; 2) myosin II filaments containing differently oriented myosin head domains, which could ease reorientation by changing the myosin head binding to the actin filament. However, the observed increase of K_{tor} with decreasing ATP concentrations could be due to the binding of multiple myosin heads simultaneously to an actin filament. This rotational flexibility is likely to allow myosin filaments to connect multiple actin filaments (40), which would be important to generate network contraction (41,42).

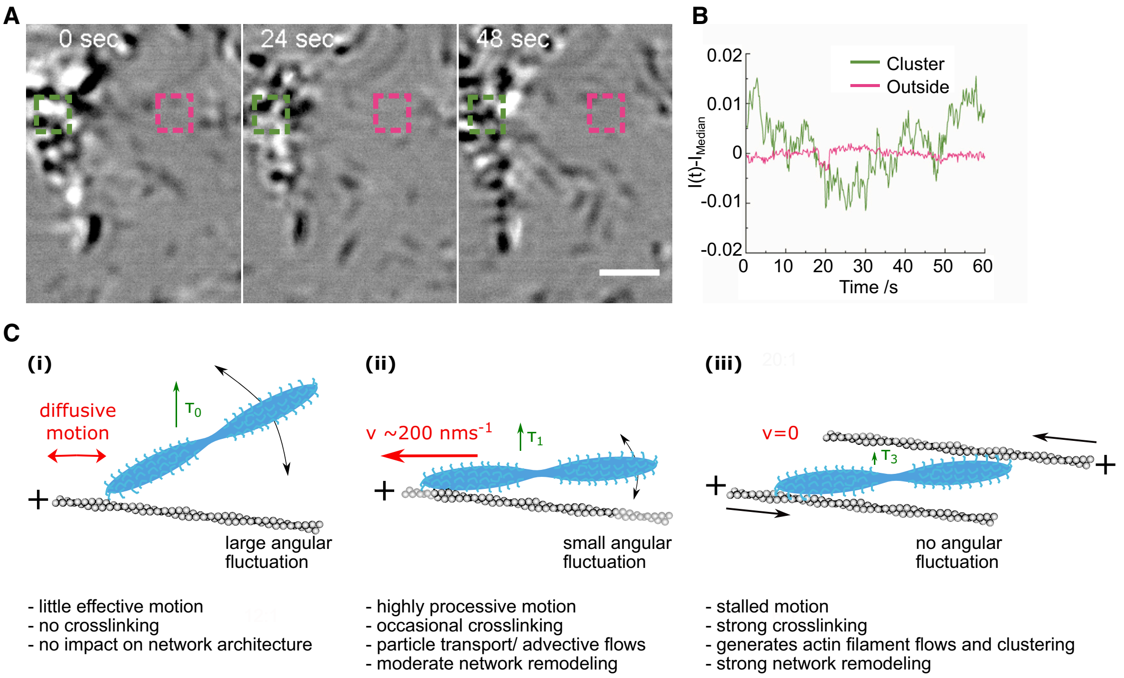

FIGURE 4 Dynamics within acto-myosin clusters. (*A*) Shown is an image sequence depicting the dynamics of myosin II filaments within an acto-myosin cluster; to make the dynamics in the cluster visible, the time median of the image sequence was subtracted from each frame; 635-nm iSCAT setup. Scale bars, 2 μm. (*B*) Shown is a graph depicting the change of interferometric contrast from the time median of the regions depicted in (*A*) inside (*green*) and outside (*magenta*) the cluster. (*C*) Shown is a schematic summarizing the different observed binding modes of myosin II filaments and their assumed effect on the acto-myosin network dynamics and organization. To see this figure in color, go online.

While we were following the network dynamics over the course of 20 min, it was striking to observe that the transition from the remodeling to the contractile state happened very suddenly at around 16 min (Video S5). This was reflected by a sudden change in the ratio of myosin II filaments showing directed motion (Fig. S3 *F*) as well as in the change in filament orientation (Fig. S3 *J*) at that time point. This indicates that gradual changes in the ensemble of myosin head domains (e.g., binding time) led to a sharp transition between regimes in which myosin II filaments either mediate a remodeling or contractile behavior of the network.

Myosin II filament flows generate transiently stable contractile zones

In acto-myosin networks that reached their contracted state, we observed the formation of contractile zones into which myosin II filaments moved continuously from multiple directions. These contractile zones were characterized by a cluster of myosin II filaments in their center holding multiple actin filaments together. Interestingly, despite the steady flow of myosin II filaments into the clusters, they did not seem to grow significantly in size or mass. After subtracting the median image of the image sequence showing a cluster region, it became evident that myosin II filaments detached once they reached the core of the cluster, preventing overall growth of the myosin II filament cluster (Fig. 4, *A* and *B*; Video S9). This could be due to the lack of free actin filaments and mechanical tension inside the clusters, forcing excess myosin filaments to leave the condensed detection zone either by detaching from the cluster or by stacking up to form three-dimensional structures above the membrane-tethered actin network as observed earlier (4,40,43).

DISCUSSION

Studying muscle myosin II filament dynamics inside a membrane-bound actin network with iSCAT microscopy has the potential to provide insights on the relation between mesoscale network dynamics and minute changes of the physicochemical properties of motor head domains in myosin II filaments. In this work, we demonstrated that changes in muscle myosin II filament dwell times and myosin filament motion can be reliably tracked within actin networks and that changes in myosin filament dynamics can be related to changes in the acto-myosin network from a remodeling to a contractile state. This is emphasized by the observation that muscle myosin II filaments move longer and more directed in the contractile state (here at t $=$ 16 min) than in the remodeling state at 100 μM ATP. These changes in muscle myosin II filament dynamics are likely to be due to a decrease in ATP concentrations over time, as previously reported (16,35). Recent theoretical studies

suggested that factors like changes in ATP concentration and mechanical load, e.g., transmitted via actin filaments, increase the processivity of ensembles of myosin II head domains and can influence myosin II filament binding dynamics (8,44). Our observations are in line with these predictions, however, the difference in ensemble size described in the theoretical models (8–48 myosin head domains) and studied here (100–250 head domains) poses limits on the comparison. The velocities reported here for myosin II filaments ($0.46 \pm 0.20\ \mu m\ s^{-1}$ at $t = 16$ min) were below velocities reported in motility assays (45) and the predicted values in (8,44), which might be due to the intermittent periods of diffusive motion of myosin II filaments and the number and flexibility of myosin head domains that can interact with actin filaments (assuming 100 myosin heads in a myosin II filament, the myosin density would be about $100/[2\pi \times 50\,\mathrm{nm} \times 700\mathrm{nm}] \approx 500\ \mu m^{-2}]$) (39). Further studies with nonmuscle myosin II filaments, which form better-defined filaments of ~30 dimeric subunits, would be needed to elucidate the mechanosensitive properties of myosin head domain ensembles and test the theoretical predictions, which mainly relied on data obtained from single myosin head domain studies (8,44). The approach introduced here could be used to test these models with data directly obtained from nonmuscle myosin II filaments in the future.

As a proof of concept, we used muscle myosin II filaments here because their size made them easily detectable in iSCAT microscopy. These should be replaced by nonmuscle myosin IIA (NMIIA) and myosin IIB filaments for more physiologically relevant studies of myosin-driven cell cortex dynamics in future experiments (46–48). Detection with iSCAT would be possible because the typical size of an NMIIA filament (~300 nm long, 29 dimers at 490 kDa) (49) would lead to a contrast ratio with an actin filament within a diffraction-limited spot of 210 ± 10 nm of $m_{myoII}/m_{F-actin} = [20\ \mathrm{dimers} \times 490\ \mathrm{kDa}]/[60\ subunits \times 42\ \mathrm{kDa}] = 3.9$ and the optical resolution of the 445-nm laser iSCAT setup would allow the detection of NMIIA filaments as elongated structures.

Given the large number of myosin head domains within a single myosin II filament, we would expect complex binding behavior with multiple characteristic attachment times. Our data, however, show that myosin II filament binding is determined by three major timescales: τ_{off0} in the range of 0.3–0.4 s, τ_{off1} in the range of 2–3 s, and τ_{off2} in the 10-s range. The shortest timescale likely corresponds to the binding and unbinding of a single myosin head domain (50). Considering the dumbbell structure of myosin II filaments, we attribute τ_{off1} to the binding of several myosin head domains at one side of the dumbbell, whereas τ_{off2} would represent the simultaneous binding of myosin heads from both sides. This model would explain the improved alignment of myosin filaments at low ATP concentrations. As the trailing end of a myosin filament turns less off axis than at high ATP concentrations, it is less likely to encounter another actin filament and form a cross-link. Therefore, we observed a reduction of long dwell times with short run lengths at low ATP concentrations. Once the myosin filaments reach the plus end of an actin filament, they could contribute to the condensation of actin filaments into clusters by catching and pulling adjacent actin filaments, leading to the formation of contractile zones in more-crowded regions of the actin filament network (3,51,52). The effect of myosin II filaments cross-linking multiple actin filaments on network contractility and entropy production was also reported by the Murrell group (42).

The large dynamic range of iSCAT microscopy allowed us to reveal the dynamics within acto-myosin clusters. Interestingly, myosin II filaments continuously moved into myosin clusters and, within these clusters, eventually detached upon reaching the clusters' center, which resulted in no or only limited growth of clusters over time. This would imply that myosin filaments inside clusters are not necessarily in a jammed state and that the turnover of myosin II filaments could contribute to the formation of constitutively active contractile zones. Taken together, our observations of myosin dynamics underline the notion that myosin II filaments can act as motors and cross-linkers, which is important to drive clustering of acto-myosin networks, and that a relatively small number of long-binding, cross-linking myosin II filaments can mediate the transition from the remodeling to the contractile state (Fig. 4 *C*).

SUPPORTING MATERIAL

Supporting Material can be found online at https://doi.org/10.1016/j.bpj.2020.02.025.

AUTHOR CONTRIBUTIONS

D.V.K. conceived, designed, and performed experiments; purified proteins; analyzed data; and wrote the manuscript. N.H. helped in the experiments, analyzed data, and wrote the manuscript. L.S.M. wrote the tracking code and performed the analysis. G.Y. and A.F. built the iSCAT microscopes. M.P. advised in the development of the tracking code and reviewed/edited the manuscript. P.K. conceived experiments and reviewed/edited the manuscript. S.M. conceived experiments and reviewed/edited the manuscript.

ACKNOWLEDGMENTS

D.V.K. thanks Madan Rao (National Center for Biological Sciences) and Kabir Husain (University of Chicago) for instructive discussions and comments on the manuscript, Mohan Balasubramanian (Warwick Medical School) for material support and instructive discussions, and the Company of Biologists for supporting this work with a Travelling fellowship. N.H. thanks James Sellers and Yasuharu Takagi from the National Institutes of Health for providing the actin.

D.V.K. was funded as a Campus Fellow at NCBS, later as a postdoctoral fellow in the Balasubramanian lab by ERC-2014-ADG No 671083, and then as an assistant professor in the Wellcome-Warwick Quantitative Biomedical Program by Wellcome Trust grant RMRCB0058. N.H. was supported by a research fellowship of the German Research Foundation

(DFG), grant HU 2462/1-1, and later by a DFG return grant HU 2462/3-1. L.S.M. was supported by Leverhulme Trust grant RPG-2016-260. G.Y. was supported by a Zvi and Ofra Meitar Magdalen Graduate Scholarship; P.K. was supported by a Starting Investigator Grant of the European Research Council (ERC; Nanoscope, 337577). S.M. and this work were supported by a JC Bose Fellowship from the Department of Science and Technology and a Margadarshi Fellowship IA/M/15/1/502018 from Department of Biotechnology - Wellcome Trust, India Alliance.

REFERENCES

1. Köster, D. V., and S. Mayor. 2016. Cortical actin and the plasma membrane: inextricably intertwined. *Curr. Opin. Cell Biol.* 38:81–89.
2. Koenderink, G. H., and E. K. Paluch. 2018. Architecture shapes contractility in actomyosin networks. *Curr. Opin. Cell Biol.* 50:79–85.
3. Ideses, Y., A. Sonn-Segev, ..., A. Bernheim-Groswasser. 2013. Myosin II does it all: assembly, remodeling, and disassembly of actin networks are governed by myosin II activity. *Soft Matter.* 9:7127–7137.
4. Köster, D. V., K. Husain, ..., S. Mayor. 2016. Actomyosin dynamics drive local membrane component organization in an in vitro active composite layer. *Proc. Natl. Acad. Sci. USA.* 113:E1645–E1654.
5. Bidone, T. C., W. Jung, ..., T. Kim. 2017. Morphological transformation and force generation of active cytoskeletal networks. *PLoS Comput. Biol.* 13:e1005277.
6. Albert, P. J., T. Erdmann, and U. S. Schwarz. 2014. Stochastic dynamics and mechanosensitivity of myosin II minifilaments. *New J. Phys.* 16:093019.
7. Rastogi, K., M. S. Puliyakodan, ..., R. Elangovan. 2016. Maximum limit to the number of myosin II motors participating in processive sliding of actin. *Sci. Rep.* 6:32043.
8. Erdmann, T., K. Bartelheimer, and U. S. Schwarz. 2016. Sensitivity of small myosin II ensembles from different isoforms to mechanical load and ATP concentration. *Phys. Rev. E.* 94:052403.
9. Kukura, P., H. Ewers, ..., V. Sandoghdar. 2009. High-speed nanoscopic tracking of the position and orientation of a single virus. *Nat. Methods.* 6:923–927.
10. Young, G., and P. Kukura. 2019. Interferometric scattering microscopy. *Annu. Rev. Phys. Chem.* 70:301–322.
11. Spudich, J. A., and S. Watt. 1971. The regulation of rabbit skeletal muscle contraction. I. Biochemical studies of the interaction of the tropomyosin-troponin complex with actin and the proteolytic fragments of myosin. *J. Biol. Chem.* 246:4866–4871.
12. Pollard, T. D. 1982. Myosin purification and characterization. *Methods Cell Biol.* 24:333–371.
13. Shrivastava, R., D. Köster, ..., M. Neerathilingam. 2015. Tailor-made ezrin actin binding domain to probe its interaction with actin in-vitro. *PLoS One.* 10:e0123428.
14. Lewis, B. A., and D. M. Engelman. 1983. Lipid bilayer thickness varies linearly with acyl chain length in fluid phosphatidylcholine vesicles. *J. Mol. Biol.* 166:211–217.
15. Nye, J. A., and J. T. Groves. 2008. Kinetic control of histidine-tagged protein surface density on supported lipid bilayers. *Langmuir.* 24:4145–4149.
16. Smith, D., F. Ziebert, ..., J. Käs. 2007. Molecular motor-induced instabilities and cross linkers determine biopolymer organization. *Biophys. J.* 93:4445–4452.
17. Ortega-Arroyo, J., and P. Kukura. 2012. Interferometric scattering microscopy (iSCAT): new frontiers in ultrafast and ultrasensitive optical microscopy. *Phys. Chem. Chem. Phys.* 14:15625–15636.
18. Cole, D., G. Young, ..., P. Kukura. 2017. Label-free single-molecule imaging with numerical-aperture-shaped interferometric scattering microscopy. *ACS Photonics.* 4:211–216.
19. Li, H., T. Shen, ..., X. Huang. 2009. Automated actin filament segmentation, tracking and tip elongation measurements based on open active contour models. *Proc. IEEE Int. Symp. Biomed. Imaging.* 2009:1302–1305.
20. Smith, M. B., H. Li, ..., D. Vavylonis. 2010. Segmentation and tracking of cytoskeletal filaments using open active contours. *Cytoskeleton (Hoboken).* 67:693–705.
21. Meijering, E., M. Jacob, ..., M. Unser. 2004. Design and validation of a tool for neurite tracing and analysis in fluorescence microscopy images. *Cytometry A.* 58:167–176.
22. Ortega Arroyo, J., D. Cole, and P. Kukura. 2016. Interferometric scattering microscopy and its combination with single-molecule fluorescence imaging. *Nat. Protoc.* 11:617–633.
23. Woody, M. S., J. H. Lewis, ..., E. M. Ostap. 2016. MEMLET: an easy-to-use tool for data fitting and model comparison using maximum-likelihood estimation. *Biophys. J.* 111:273–282.
24. Mosby, L., M. Polin, and D. V. Köster. 2020. A Python based automated tracking routine for myosin II filaments. *arXiv*, arXiv:2001.10035 https://arxiv.org/abs/2001.10035.
25. Bertin, E., and S. Arnouts. 1996. SExtractor: software for source extraction. *Astron. Astrophys. Suppl. Ser.* 117:393–404.
26. Barbary, K. 2016. SEP: source extractor as a library. *J. Open Source Softw.* 1:58.
27. Barbary, K. 2018. sep Documentation- Release 1.0.3. https://sep.readthedocs.io/en/v1.0.x/.
28. Young, G., N. Hundt, ..., P. Kukura. 2018. Quantitative mass imaging of single biological macromolecules. *Science.* 360:423–427.
29. AL-Khayat, H. A. 2013. Three-dimensional structure of the human myosin thick filament: clinical implications. *Glob. Cardiol. Sci. Pract.* 2013:280–302.
30. Carlier, M. F. 1991. Actin: protein structure and filament dynamics. *J. Biol. Chem.* 266:1–4.
31. Pardee, J. D., and J. A. Spudich. 1982. Purification of muscle actin. *Methods Cell Biol.* 24:271–289.
32. Grover, R., J. Fischer, ..., S. Diez. 2016. Transport efficiency of membrane-anchored kinesin-1 motors depends on motor density and diffusivity. *Proc. Natl. Acad. Sci. USA.* 113:E7185–E7193.
33. Chakraborty, I., and Y. Roichman. 2019. Two coupled mechanisms produce Fickian, yet non-Gaussian diffusion in heterogeneous media. *arXiv*, arXiv:1909.11364 https://arxiv.org/abs/1909.11364.
34. Ditlev, J. A., A. R. Vega, ..., M. K. Rosen. 2019. A composition-dependent molecular clutch between T cell signaling condensates and actin. *eLife.* 8:e42695.
35. Vogel, S. K., Z. Petrasek, ..., P. Schwille. 2013. Myosin motors fragment and compact membrane-bound actin filaments. *eLife.* 2:e00116.
36. Murrell, M. P., and M. L. Gardel. 2012. F-actin buckling coordinates contractility and severing in a biomimetic actomyosin cortex. *Proc. Natl. Acad. Sci. USA.* 109:20820–20825.
37. Billington, N., D. J. Revill, ..., P. J. Knight. 2014. Flexibility within the heads of muscle myosin-2 molecules. *J. Mol. Biol.* 426:894–907.
38. Burgess, S. A., M. L. Walker, ..., J. Trinick. 1997. Flexibility within myosin heads revealed by negative stain and single-particle analysis. *J. Cell Biol.* 139:675–681.
39. Winkelmann, D. A., L. Bourdieu, ..., A. Libchaber. 1995. Flexibility of myosin attachment to surfaces influences F-actin motion. *Biophys. J.* 68:2444–2453.
40. Melli, L., N. Billington, ..., J. R. Sellers. 2018. Bipolar filaments of human nonmuscle myosin 2-A and 2-B have distinct motile and mechanical properties. *eLife.* 7:1–25.
41. Alvarado, J., M. Sheinman, ..., G. H. Koenderink. 2013. Molecular motors robustly drive active gels to a critically connected state. *Nat. Phys.* 9:591–597.
42. Seara, D. S., V. Yadav, ..., M. P. Murrell. 2018. Entropy production rate is maximized in non-contractile actomyosin. *Nat. Commun.* 9:4948.
43. Das, A., A. Bhat, ..., M. Rao. 2019. Assemblies of F-actin and myosin-II minifilaments: steric hindrance and stratification at the membrane cortex. *bioRxiv* https://doi.org/10.1101/656082v1.

44. Stam, S., J. Alberts, …, E. Munro. 2015. Isoforms confer characteristic force generation and mechanosensation by myosin II filaments. *Biophys. J.* 108:1997–2006.

45. Kron, S. J., and J. A. Spudich. 1986. Fluorescent actin filaments move on myosin fixed to a glass surface. *Proc. Natl. Acad. Sci. USA.* 83:6272–6276.

46. Shutova, M. S., S. B. Asokan, …, T. M. Svitkina. 2017. Self-sorting of nonmuscle myosins IIA and IIB polarizes the cytoskeleton and modulates cell motility. *J. Cell Biol.* 216:2877–2889.

47. Beach, J. R., and J. A. Hammer, III. 2015. Myosin II isoform co-assembly and differential regulation in mammalian systems. *Exp. Cell Res.* 334:2–9.

48. Heuzé, M. L., G. H. N. Sankara Narayana, …, B. Ladoux. 2019. Myosin II isoforms play distinct roles in *adherens* junction biogenesis. *eLife.* 8:e46599.

49. Billington, N., A. Wang, …, J. R. Sellers. 2013. Characterization of three full-length human nonmuscle myosin II paralogs. *J. Biol. Chem.* 288:33398–33410.

50. Erdmann, T., and U. S. Schwarz. 2012. Stochastic force generation by small ensembles of myosin II motors. *Phys. Rev. Lett.* 108:188101.

51. Soares e Silva, M., M. Depken, …, G. H. Koenderink. 2011. Active multistage coarsening of actin networks driven by myosin motors. *Proc. Natl. Acad. Sci. USA.* 108:9408–9413.

52. Linsmeier, I., S. Banerjee, …, M. P. Murrell. 2016. Disordered actomyosin networks are sufficient to produce cooperative and telescopic contractility. *Nat. Commun.* 7:12615.

Biophysical Journal, Volume *118*

Supplemental Information

Myosin II Filament Dynamics in Actin Networks Revealed with Interferometric Scattering Microscopy

Lewis S. Mosby, Nikolas Hundt, Gavin Young, Adam Fineberg, Marco Polin, Satyajit Mayor, Philipp Kukura, and Darius V. Köster

Myosin II filament dynamics in actin networks revealed with interferometric scattering microscopy

Lewis S. Mosby[1,2]*, Nikolas Hundt[3,ɫ,]*, Gavin Young[3], Adam Fineberg[3], Marco Polin[1,2], Satyajit Mayor[4,5,#], Philipp Kukura[3#], Darius V. Köster[1,6#]

1 Centre for Mechanochemical Cell Biology, University of Warwick, Coventry CV4 7AL, UK

2 Physics Department, University of Warwick, Coventry CV4 7AL, UK

3 Physical and Theoretical Chemistry Laboratory, Department of Chemistry, University of Oxford, South Parks Road, Oxford OX1 3QZ, UK

4 National Centre for Biological Sciences, Tata Institute for Fundamental Research, GKVK, Bellary Road, Bangalore 560065, India

5 Institute for Stem Cell Biology and Regenerative Medicine, Bangalore 560065, India

6 Division of Biomedical Sciences, Warwick Medical School, University of Warwick, Coventry CV4 7AL, UK

* equal contribution

#corresponding authors: mayor@ncbs.res.in, philipp.kukura@chem.ox.ac.uk, d.koester@warwick.ac.uk

ɫ current address: Department of Cellular Physiology, Biomedical Center, Ludwig-Maximilians-Universität München, Großhaderner Str. 9, 82152 Planegg/Martinsried, Germany

Supplementary material

Detection of myosin II filaments

We use two criteria for the detection of a bound myosin filament: particles have to be above an intensity threshold, and they have to display a minimal area characteristic for a single myosin filament oriented along the focal plane. This ensures that the probability to detect a myosin filament that is diffusing freely and not binding to F-actin is very low.

Considering an unbound myosin filament, it would move out of radius *r* = 200nm sphere within <10 ms, which would not generate enough signal to be considered by our tracking algorithm.

This estimate is based on taking the average time for a diffusing particle to travel from the center to a point on a sphere with radius *r*: $\bar{t} = \frac{r^2}{6D}$

And with $D = \frac{\mathrm{k_B}T}{6\,\pi\gamma R}$, where $\gamma = 0.89\ 10^{-3}\ \mathrm{kg\,s^{-1}\,m^{-1}}$ is the viscosity of water at 23 °C and $R\ =\ 250\ \mathrm{nm}$ is the radius of a sphere to represent a myosin filament, we get

$$\bar{t} = \frac{(0.2\ \mu\mathrm{m})^2}{6\ \times\ 1.48\ \mu\mathrm{m}^2\mathrm{s}^{-1}} = 6.7\ \mathrm{ms}$$

And if we take into account the elongated shape of myosin, with the long semi-axis $b\ =\ 300\ \mathrm{nm}$, a short semi-axis $a\ =\ 50\ \mathrm{nm}$ and $D = \frac{\mathrm{k_B}T\ \ln(2b/a)}{4\,\pi\gamma\ b}$, we get

$$\bar{t} = \frac{(0.2\ \mu\mathrm{m})^2}{6\ \times 3.31\ \mu\mathrm{m}^2\mathrm{s}^{-1}} = 3\ \mathrm{ms}$$

Mobility of actin filaments and slippage of myosin II filaments on supported lipid bilayers

Here, we are considering the possibility that the motor action of a myosin II filament would lead to a slippage of the actin filament resulting in a reduced net velocity of the myosin filament. For this, we compare the friction of the actin filament created by the actin-membrane linker and compare it to the friction that myosin II filaments experience in aqueous solutions.

Similar to considerations described in (30), we can estimate the frictional force on the actin-membrane linker by using the Einstein-Smoluchowski relation, where the drag coefficient is related to its diffusivity D_{HKE} (= 1 $\mu m^2 s^{-1}$) by:

$$\gamma_{HKE} = \frac{\mathrm{k_B}T}{D_{HKE}}$$

The drag force on a single actin filament could then be estimated as:

$$F_{actin} = \ L_{actin}\, \rho_{HKE}\gamma_{HKE}\ v_{actin} = L_{actin}\, \rho_{HKE} \frac{\mathrm{k_B}T}{D_{HKE}}\ v_{actin}$$

If we consider an actin filament of L_{actin} = 5 µm length, a linear membrane-actin linker density of ρ_{HKE} = 30 μm^{-1} and a maximum velocity of v_{actin} = 400 nm s^{-1} (if the myosin would stand still and move actin at maximum speed) the drag force would be

$$F_{actin}^{max} = 0.25\ \mathrm{pN}$$

with k_B = 1.38 10^{-23} JK^{-1} and T = 295K.

In contrast, the drag force that acts on a myosin filament when it moves through an aqueous solution, can be estimated using the drag coefficient of a cylindrical object of length L and radius r moving parallel to the surface in a distance h:

$$\gamma_{\mathrm{myo}} = \frac{2\pi\eta \mathrm{L_{myo}}}{\ln(^{2\mathrm{h}}/_{\mathrm{r_{myo}}})}$$

And the drag force for the myosin filament would be

$$\mathrm{F_{myo}} = \gamma_{\mathrm{myo}}\mathrm{v_{myo}} = \frac{2\pi\eta \mathrm{L_{myo}}}{\ln(^{2\mathrm{h}}/_{\mathrm{r_{myo}}})}\ \mathrm{v_{myo}}$$

With η = 10^{-3} Pa s (water), L_{myo} = 550 nm, h = 60 nm, r_{myo} = 30 nm, and v_{myo} = 400 nm s^{-1} the drag force is

$$\mathrm{F_{myo}} = 1\ \mathrm{fN}$$

That means that the drag on the myosin filament is 250 times smaller than the drag on the actin filament. This explains why myosin filaments move along single actin filaments without causing any significant displacement of actin filaments and why changes in actin filament organization only occur when myofilaments bind to multiple actin filaments, resulting to higher friction on myosin II filament motion and force transduction to the actin network.

Video legends, supplementary figures and tables

Video 1 Movie recorded with a 445 nm laser iSCAT system showing the formation of an SLB by landing and fusion of single uni-lamellar vesicles on a glass slide. The reduced contrast at the end of the video indicates the presence of the SLB as it smoothens the glass roughness.

Video 2 Movie showing the increase in interferometric scattering when an actin filament lands on top of another (Fig. S1E, F)

Video 3 Movie showing diffusion of a 1 µm long actin filament within a membrane-bound actin network (removed by median filtering) (Fig. 1E, F and Fig. S1H)

Video 4 Movie showing diffusion of a 4 µm long actin filament within a membrane-bound actin network (removed by median filtering) (Fig. 1G and Fig. S1I)

Video 5 Movie showing the transition of the acto-myosin network from a remodeling to a contractile state upon depletion of ATP over time (Fig. S2A, B).

Video 6 Movie showing the release of myosin II filaments from actin after addition of fresh ATP (100 µM) indicating that the contractile state of myosin II filaments in this set up is reversible.

Video 7 Movie showing myosin II filament motion along actin filaments (Fig. S2C, D). Scale bar 1 µm.

Video 8 Movie showing tracking of a single myosin filament (Fig. 3A, B; S3 A-C). Scale bar 1 µm.

Video 9 Movie showing the flow of myosin II filaments into and within an acto-myosin cluster after subtraction of the time median (Fig. 4 A, B). Scale bar 2 µm.

Figure S1

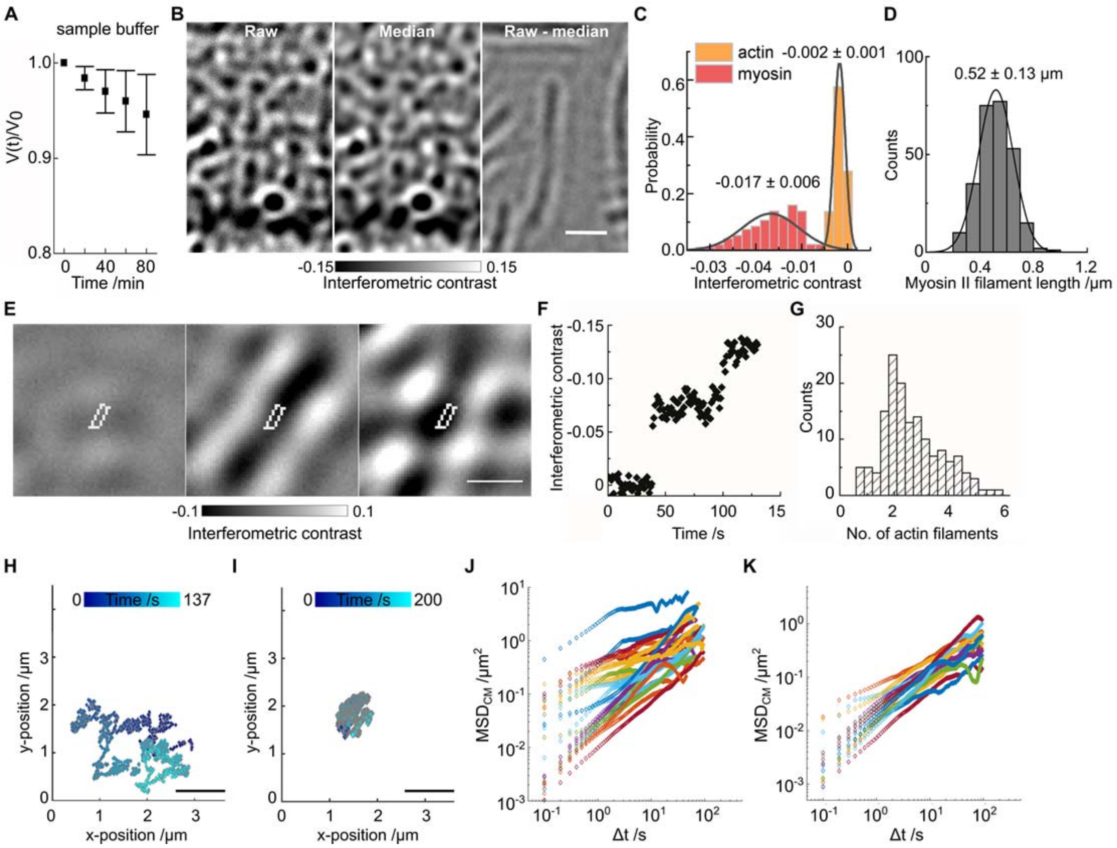

Figure S1, Detection and identification of actin and myosin II filaments: A) Graph illustrating the typical loss of buffer due to evaporation from the experimental chambers at room temperature; data points represent average ± standard deviation of three independent experiments. **B)** Example of median filter to visualize individual actin filaments landing afresh or moving within a static actin network; the median of the image sequence is subtracted from the raw images; 445 nm laser set-up, scale bar: 1 µm. **C)** Histogram of actin filament and myosin II filament contrast measurements with the 635 nm laser iSCAT set-up; actin filaments: N = 352 measurements along N_{fil} = 15 filaments and N_{myoII} = 94; black lines depict fit of normal distribution. **D)** Histogram of myosin II filament length measurements with the 635 nm laser iSCAT set-up; black line depicts fit of normal distribution; N = 269. **E)** Image sequence of an actin filament landing on top of another imaged with the 445 nm iSCAT setup. **F)** Corresponding measurement of the interferometric contrast of the region depicted by the white box in (E) showing a stepwise increase of the signal corresponding to one actin filament landing on top of another. **G)** Histogram of the actin layer thickness in a typical experiment based on background-subtracted, local interferometric contrast measurements divided by the average contrast value of an individual actin filament (C) (N=149). **H)** Center of mass trajectory of a 1 µm long actin filament (Fig. 1E, F; Video 3) over time (from dark blue to cyan: 0- 137s), scale bar: 1 µm. **I)** Center of mass trajectory of a 4 µm long actin filament (Fig 1G; Video 4) confined by the surrounding actin network (from dark blue to cyan: 0-200 s), scale bar: 1 µm. **J, K)** Combined plot of MSDs of actin filaments shorter than 1.5 µm (**J**) or longer (**K**).

Figure S2

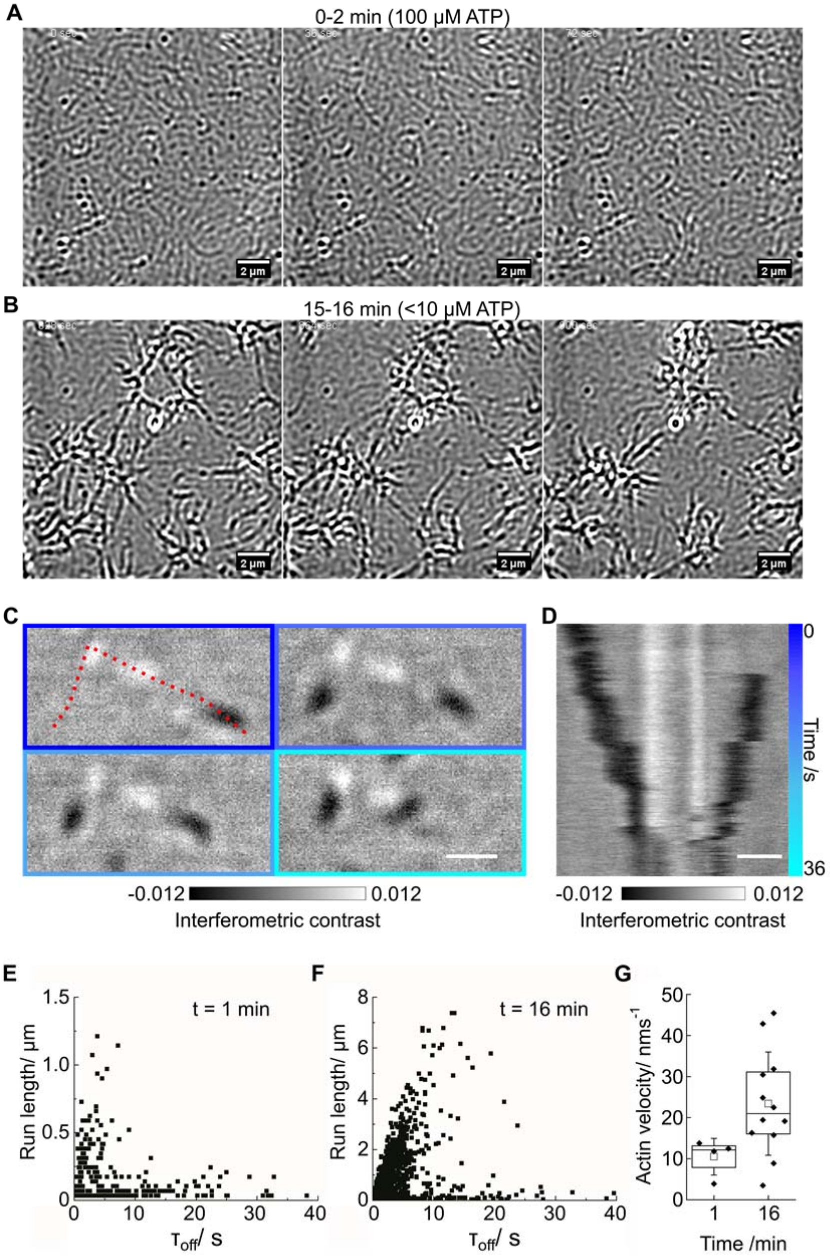

Figure S2, Myosin II filament dynamics: A, B) Image sequence depicting the dynamics of a membrane bound acto-myosin network at (**A**) the start of the experiment, in the remodeling state at 100 µM ATP and (**B**) after 16 min, when the network becomes contractile; images were acquired with the 445 nm laser iSCAT setup; scale bar: 2 µm. **C)** Image sequence showing the run of two myosin II filaments (black) on actin filaments; 635 nm laser iSCAT setup, scale bar: 1 µm. **D)** Corresponding kymograph obtained along the red lines in (C), black regions correspond to myosin II filaments that are moving along actin filaments; time color bar: 36 s, scale bar: 1 µm. **E)** Plot of run length vs binding time of myosin II filaments at t = 1 min (100 µM ATP) (Fig. 2E). **F)** Plot of run length vs binding time of myosin II filaments at t = 16 min (Fig. 2H). **G)** Boxplot depicting the velocity of actin filaments in single regions of the acto-myosin network indicating a very limited impact of actin filament displacement on the myosin II filament velocity measurements.

Figure S3

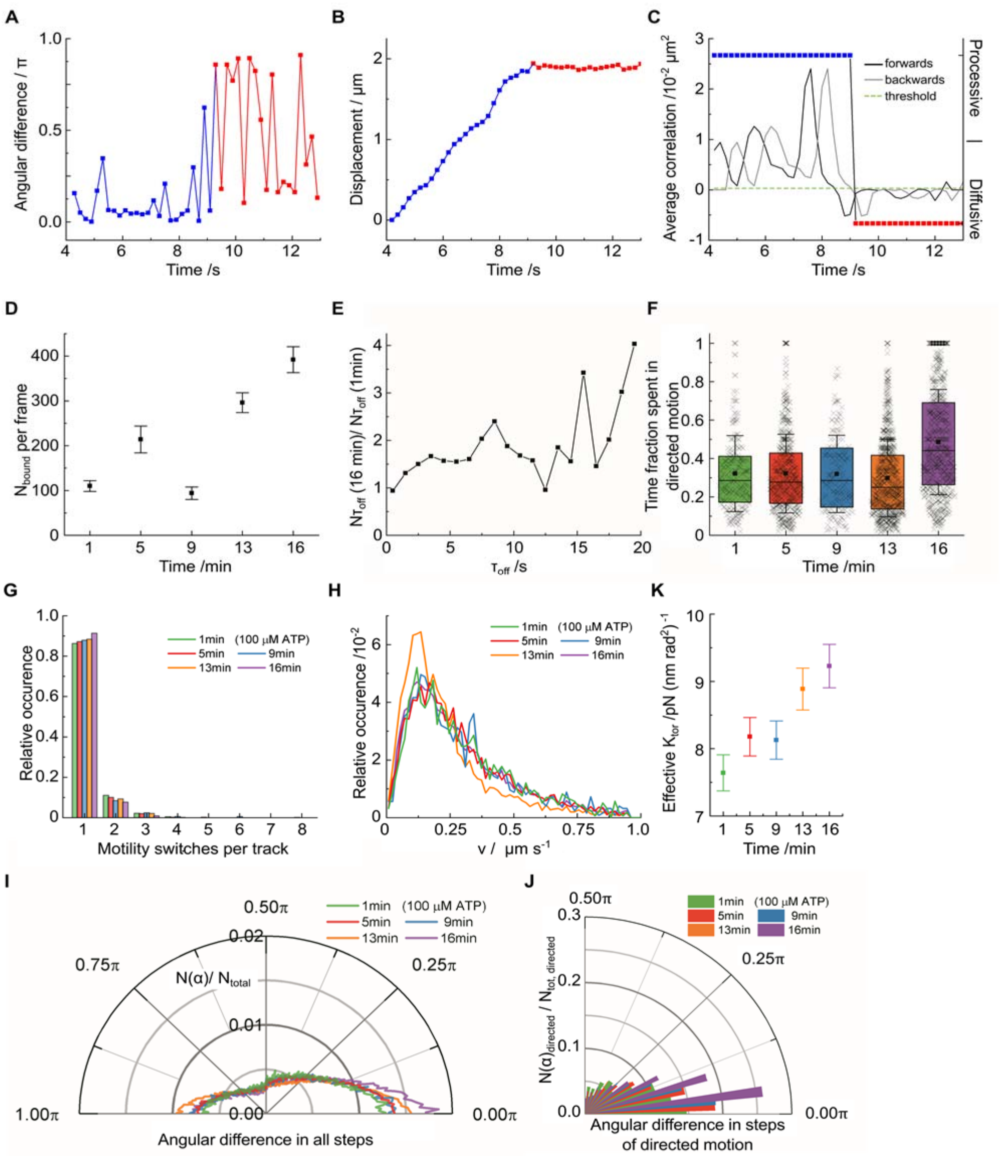

Figure S3, Myosin II filament single-particle tracking: A-C) Example of the routine to classify track segments of directed or random motion: based on the tracking data, the angular difference between particle orientation and direction of motion (**A**), the straight-line distance from the particle's origin (**B**) and the average correlation in displacements between adjacent timesteps (forward: $C_+ = \frac{\boldsymbol{dr}_{t+2\Delta t}\cdot\boldsymbol{dr}_{t+\Delta t} + \boldsymbol{dr}_{t+\Delta t}\cdot\boldsymbol{dr}_t}{2}$, backward: $C_- = \frac{\boldsymbol{dr}_{t-2\Delta t}\cdot\boldsymbol{dr}_{t-\Delta t} + \boldsymbol{dr}_{t-\Delta t}\cdot\boldsymbol{dr}_t}{2}$) were calculated (**C**) to reduce false positive detection; segments of correlation of displacement above the threshold (here 0.5 nm^2 as calculated from the average diffusivity of all myosin II filaments at t = 1 min) were classified as directed motion (blue dotted line), whereas segments below the threshold were deemed to be of random motion (red dotted line). **D)** Plot of the average (± sdv) number of automatically detected myosin II filaments per frame in the recordings at the indicated timepoints (N > 250 frames for each condition). **E)** Plot of the ratio of the numbers of detected myosin II filaments that exhibit each binding time at t=16 min (contractile state) and t = 1 min (remodeling state), indicating a 1.6-fold increase for binding times longer than 3 s; counts were normalized to the total number of detections at t = 1 min and t = 16 min. The strong fluctuations at times longer than 10s are due to the small numbers (< 10) of detected events. **F)** Box plot depicting the ratio of the duration of directed motion versus dwell time for each myosin II filament displaying directed motion at the indicated time points (N(1, 5, 9, 13, 16 min) = [221, 374, 158, 608, 640]). **G)** Histogram of the switches of each tracked myosin between directed and undirected motion at the indicated time points (sample numbers are

similar as in F); counts were divided by the total number of counts. **H)** Myosin II filament velocity distribution during directed motion based on the displacement between consecutive frames (Δt = 0.2 s) at the indicated time points of the experiment; counts were divided by the total number of counts. **I)** Radial plot depicting the angular difference α between myosin II filament orientation and its velocity vector for all detected myosin II filament steps at the indicated time points. **J)** Radial histogram plot depicting the ratio of steps at directed motion versus all detected steps for each angular difference α at indicated time points. **K)** Plot of the average (±std) effective torsional spring constant of myosin II filaments at the indicated time points; std estimation is based on the error of myosin orientation detection.

**Table S1. Summary of the experimental conditions**						
Figure	[Actin]/ nM	[CP]/ nM	$L_{F\text{-actin}}$ /µm	[Myosin II]/ nM	$[ATP]_{initial}$/ µM	λ(laser)/ nm
1B	100	0	NA	0	0	445
1C	0	0	0	20	0	445
1E-H	125	0.75	3 ± 1.4	0	100	445
2, 3	300	0	7 ± 3.5	100	100	635
4 A, B	300	0	7 ± 3.5	100	100	635
S2A, B	250	0.5	2 ± 1.4	30	100	445

Table S1, experimental conditions: List of the experimental conditions used in the examples depicted in Figs. 1–4. The table contains the concentration of G-actin, capping protein (CP), myosin II proteins and ATP as well as the average length of actin filaments attached to SLBs, which was measured before the addition of myosin II. Sample size for filament length measurements: N = 505 (Fig.1E-H), N = 151 (Fig 2, 3, 4), N = 255 (Fig S2A, B).
